# Molecular Orbital Tuning of Pentacene-Based Organic Semiconductors through *N*-Ethynylation of Dihydrodiazapentacene

**DOI:** 10.1021/jacs.4c14775

**Published:** 2025-01-21

**Authors:** Li Zhang, Yujie Zhao, Jiasheng Li, Yuang Fu, Boyu Peng, Jun Yang, Xinhui Lu, Qian Miao

**Affiliations:** a Department of Chemistry, The Chinese University of Hong Kong, Shatin, New Territories, Hong Kong, China; b State Key Laboratory of Synthetic Chemistry, The Chinese University of Hong Kong, Hong Kong, China; c Department of Physics, The Chinese University of Hong Kong, Shatin, New Territories, Hong Kong, China; d MOE Key Laboratory of Macromolecular Synthesis and Functionalization, Zhejiang University, Hangzhou 310027, China; e International Research Center for X Polymers, Zhejiang University, Hangzhou 310027, China; f Department of Polymer Science and Engineering, Zhejiang University, Hangzhou 310027, China; g Department of Chemistry, The University of Hong Kong, Hong Kong, China; h State Key Laboratory of Synthetic Chemistry, The University of Hong Kong, Hong Kong, China

## Abstract

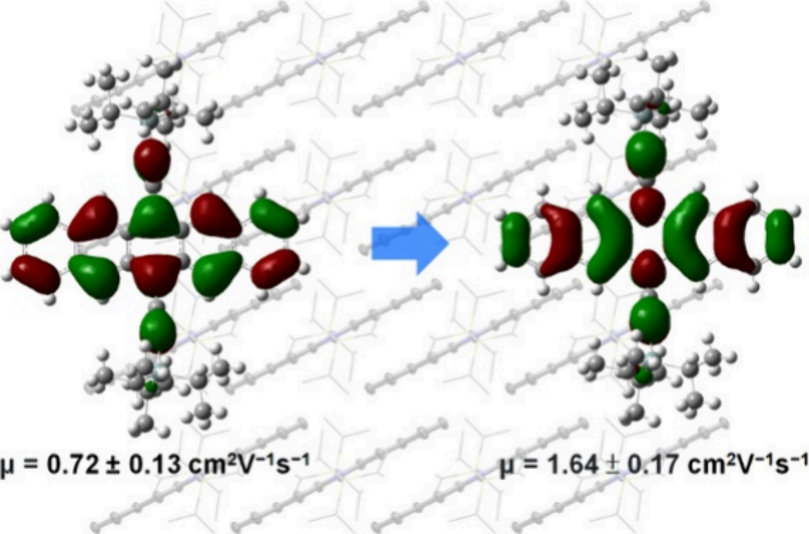

This study explores the concept of molecular orbital tuning for organic semiconductors through the use of *N,N*′-diethynylated derivatives of 6,13-dihydro-6,13-diazapentacene (**2a** and **2b**). These novel molecules maintain the same molecular geometry and π–π stacking as their parent pentacene derivatives (**1a** and **1b**), as confirmed by X-ray crystallography. However, they exhibit altered frontier molecular orbitals in terms of the phase, nodal properties, and energy levels. Theoretical calculations based on crystal structures indicate that **2a** and **2b** could significantly enhance the hole mobilities of the parent compounds by improving the hole transfer integral. Organic field-effect transistors (OFETs) of **1a** and **2a** were fabricated by using dip-coating and bar-coating methods. Both types of devices for **2a** demonstrated a hole mobility exceeding 1 cm^2^ V^–1^ s^–1^, more than twice that of the respective devices for **1a**. Additionally, unlike its pentacene parent, **2a** is transparent to visible light and exhibits significantly enhanced environmental stability against light and air, making it a promising candidate for broader applications in organic electronic devices.

## Introduction

**Figure 1:**
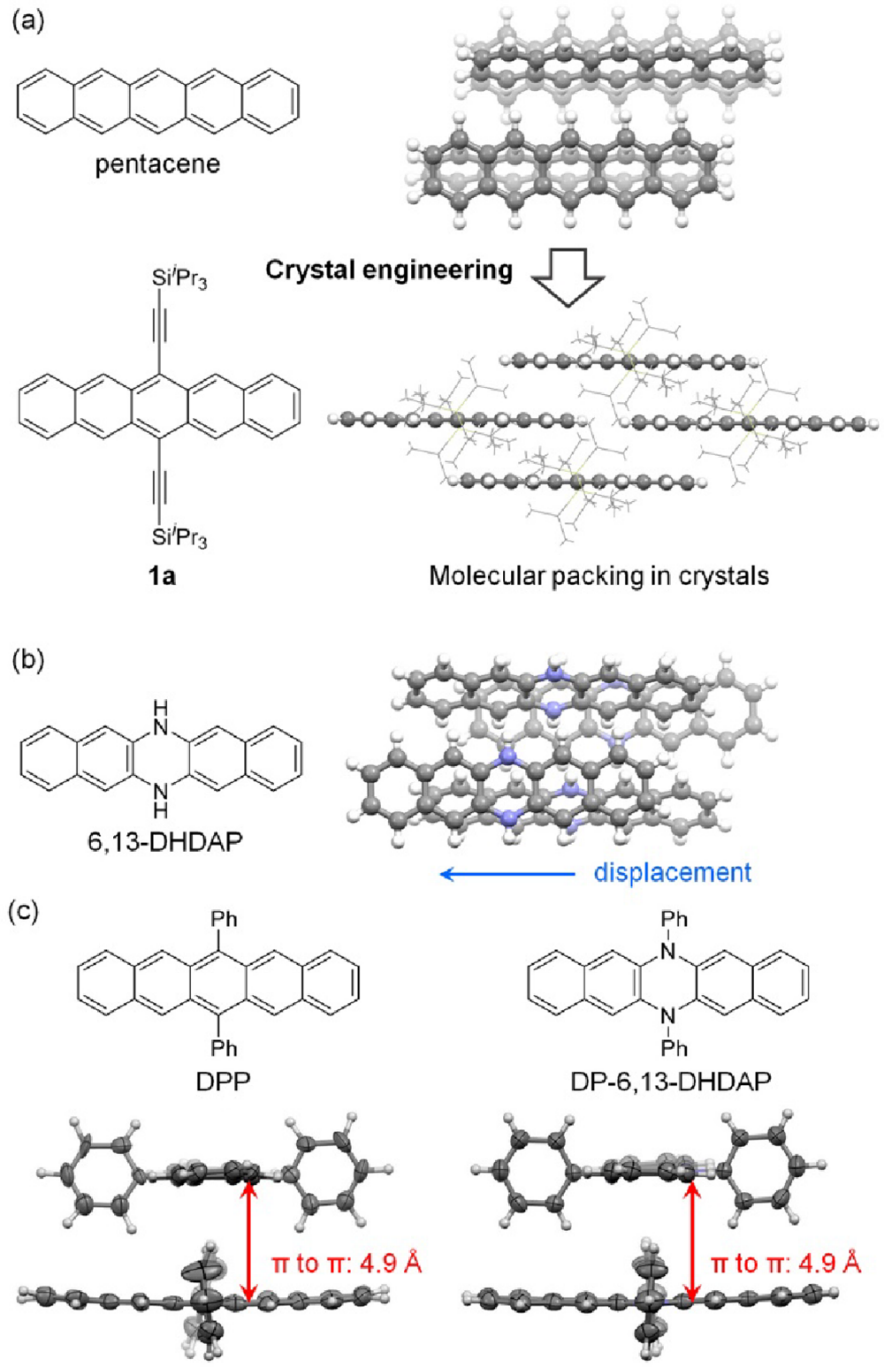
(a) Structures of pentacene and **1a** and their molecular packing in the crystals (the triisopropylsilylethynyl groups are shown with wire models, and other atoms are shown with ball and stick models). (b) Structure of 6,13-DHDAP and its molecular packing in the crystal. (c) Structure of DPP and DP-6,13-DHDAP and their molecular packing in the crystals.

Charge transfer integral and reorganization energy are crucial factors in determining the rate of charge transport in organic semiconductors,^1^ as described by Marcus theory,^2,3^ which outlines electron transfer processes. Consequently, a higher charge transfer integral and a lower reorganization energy lead to increased charge carrier mobility in organic field-effect transistors (OFETs),^4^ which are fundamental components of organic integrated circuits, playing an important role in low-cost, flexible, and wearable organic electronics.^5−7^ The charge transfer integral is governed by electronic coupling between neighboring semiconductor molecules, in a way that intimately depends on both the relative positions of interacting molecules and the phase and nodal properties of π-orbitals.^8^ Thus, it can, in principle, be enhanced by optimizing the arrangement of π-backbones or by tuning the phase and nodal characteristics of the π-orbitals. Significant efforts have been made to fine-tune the molecular packing of organic semiconductors in the solid state for high-performance OFETs.^9−13^ Such a strategy is also known as crystal engineering,^14^ which typically involves attaching different substituting groups to the same π-backbone to modify its packing in the crystals. Notably, the introduction of substituents usually does not substantially alter the phase and nodal properties of frontier π-orbitals. One of the best examples of crystal engineering for organic semiconductors in OFETs is the addition of triisopropylsilylethynyl groups to pentacene, resulting in 6,13-bis(triisopropylsilylethynyl)pentacene (**1a** in Figure 1a),^15^ a solution-processed organic semiconductor with high hole mobility.^16^ As illustrated in Figure 1a, this modification transforms the herringbone packing of pentacene into a two-dimensional (2D) π-stacking with a brickwork arrangement.

Herein, we introduce a novel strategy called molecular orbital tuning that modifies the frontier molecular orbitals of an organic semiconductor without changing its shape and molecular packing in the crystal structure. One promising candidate for this strategy is 6,13-dihydro-6,13-diazapentacene (6,13-DHDAP, shown in Figure 1b),^17^ which maintains the same planar geometry as pentacene but differs in the phase and nodal properties of its highest occupied molecular orbital (HOMO) due to the presence of two additional π-electrons.^18^ However, a detailed analysis of the crystal structures of both 6,13-DHDAP and pentacene reveals that neighboring 6,13-DHDAP molecules exhibit significant displacement along the long molecular axis relative to pentacene (Figure 1b), despite both adopting similar herringbone packing. This displacement, presumably due to the avoidance of direct overlap of the electron-rich dihydropyrazine units, disqualifies 6,13-DHDAP from effective molecular orbital tuning of pentacene. Further analysis of the crystal structures of reported derivatives of 6,13-DHDAP^19,20^ and 5,14-dihydro-5,14-diazapentacene (5,14-DHDAP) also found them unsuitable for molecular orbital tuning of pentacene-based organic semiconductors. For instance, *N*,*N′*-diphenyl-6,13-dihydro-6,13-diazapentacene (DP-6,13-DHDAP in Figure 1c) and 6,13-diphenylpentacene (DPP in Figure 1c) exhibit essentially the same molecular packing due to dominating edge-to-face interactions between the phenyl substituents and the pentacene core.^21^ However, their pentacyclic π-planes are separated by distances of up to 4.9 Å, resulting in poor semiconductor properties with low charge carrier mobility. Additionally, 6,13-bis(triisopropylsilylethynyl)-5,14-dihydro-5,14-diazapentacene adopts a 2D π-stacking with a brickwork arrangement similar to **1a**.^22^ However, the difference in overlap between the adjacent pentacyclic π-planes is not negligible, and more importantly, the disorder in the arrangement of molecules introduces uncertainty to the molecular packing (Figure S10 in the Supporting Information).

**Figure 2:**
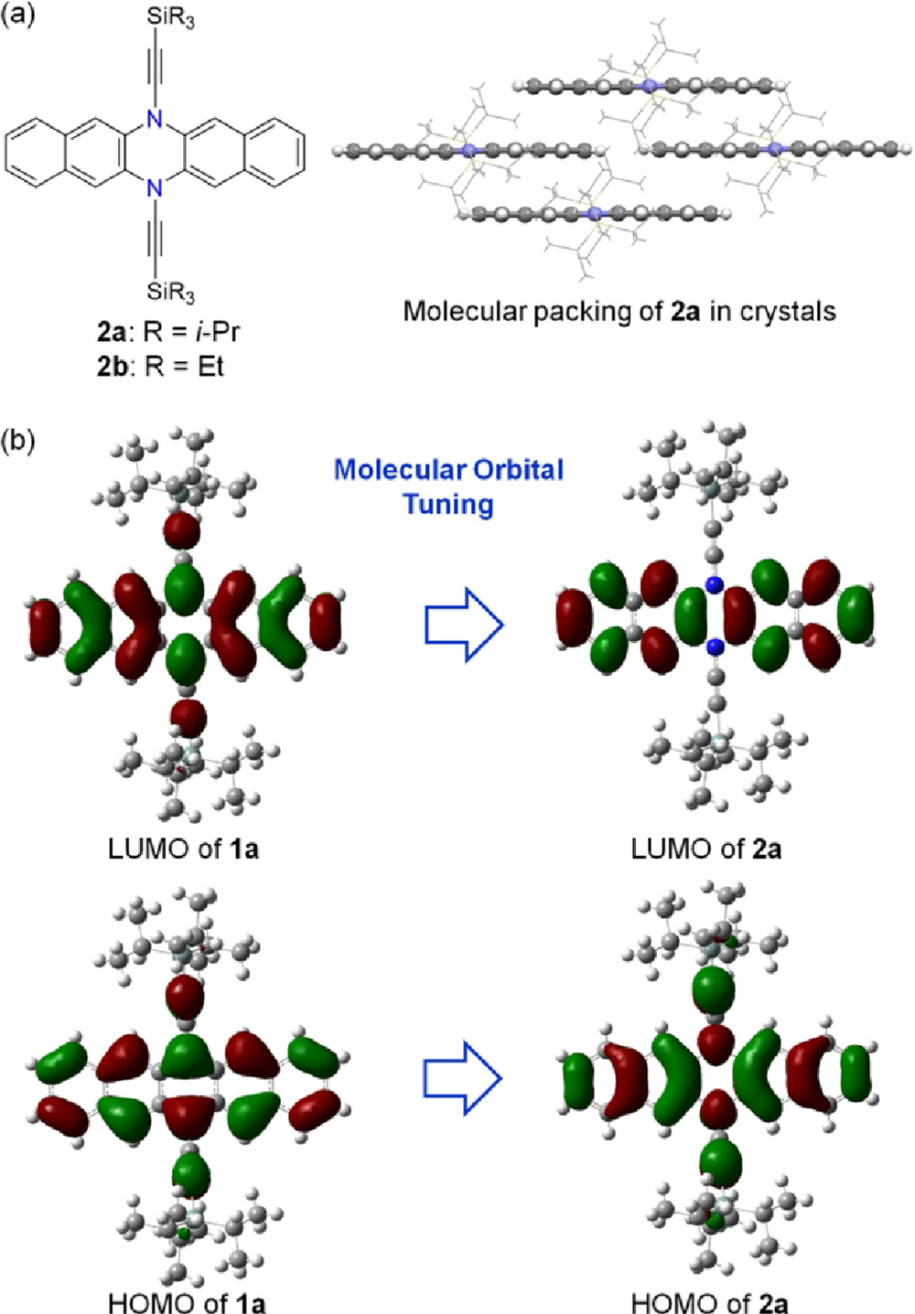
(a) Structures of **2a/b** and the molecular packing of **2a** in the crystal (the triisopropylsilylethynyl groups are shown with wire models, and other atoms are shown with ball and stick models). (b) Frontier molecular orbitals of **1a** and **2a** calculated at the B3LYP level of density functional theory (DFT) with the 6-311++G(d,p) basis set.

In this study, we demonstrate molecular orbital tuning using *N,N*′-diethynylated 6,13-DHDAPs (**2a**/**b**, shown in Figure 2a). These compounds are distinct from previously reported ethynylated *N*-heteroacenes,^23−27^ as the ethynyl groups are bonded to nitrogen atoms rather than carbon atoms. As shown in Figure 2a, compound **2a** maintains the same two-dimensional π-stacking with a brickwork arrangement as **1a**, driven by the bulky triisopropylsilylethynyl groups. On the other hand, the frontier molecular orbitals of **2a** differ from that of **1a** in both phase and nodal properties as shown in Figure 2b due to the presence of two additional π-electrons. Herein, we report the synthesis, electronic properties, and crystal structures of **2a**/**b**, along with experimental and computational studies of their semiconductor properties compared to the corresponding ethynylated pentacenes. Notably, **2a** and **2b** demonstrated field-effect mobilities in OFETs that are more than double those of **1a** and **1b**, respectively, highlighting the effectiveness of the molecular orbital tuning strategy.

## Results and Discussion

**Scheme 1:**
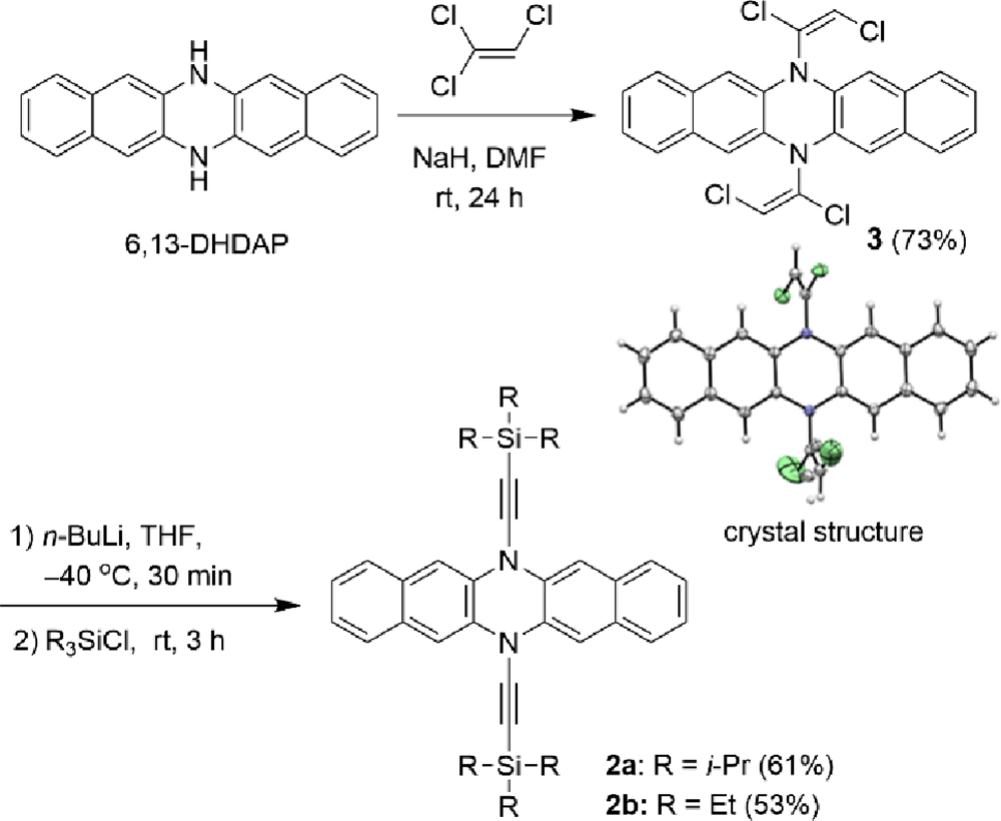
Synthesis of **2a** and **2b**

Scheme 1 illustrates the synthesis of compounds **2a** and **2b**, which builds on the previously reported method for ynamides using trichloroethylene as a two-carbon synthon.^28^ Unlike the original ynamide synthesis, which used a weak base such as Cs_2_CO_3_, our approach employed sodium hydride to deprotonate 6,13-DHDAP and eliminate HCl from trichloroethylene, generating dichloroacetylene *in situ*. The subsequent addition of the 6,13-DHDAP anion to dichloroacetylene produced compound **3** in a yield of 73%. The *E* configuration of the C–C double bonds in **3** was confirmed by X-ray crystallography. Dehalogenation of compound **3** with *n*-BuLi, involving the elimination of HCl and halogen-lithium exchange, resulted in an alkynide intermediate. This intermediate was then trapped with trialkylsilane chloride, yielding compounds **2a** and **2b** as white solids. Both **2a** and **2b** demonstrated good solubility in common organic solvents such as hexane, toluene, CH_2_Cl_2_, and CHCl_3_.

**Figure 3:**
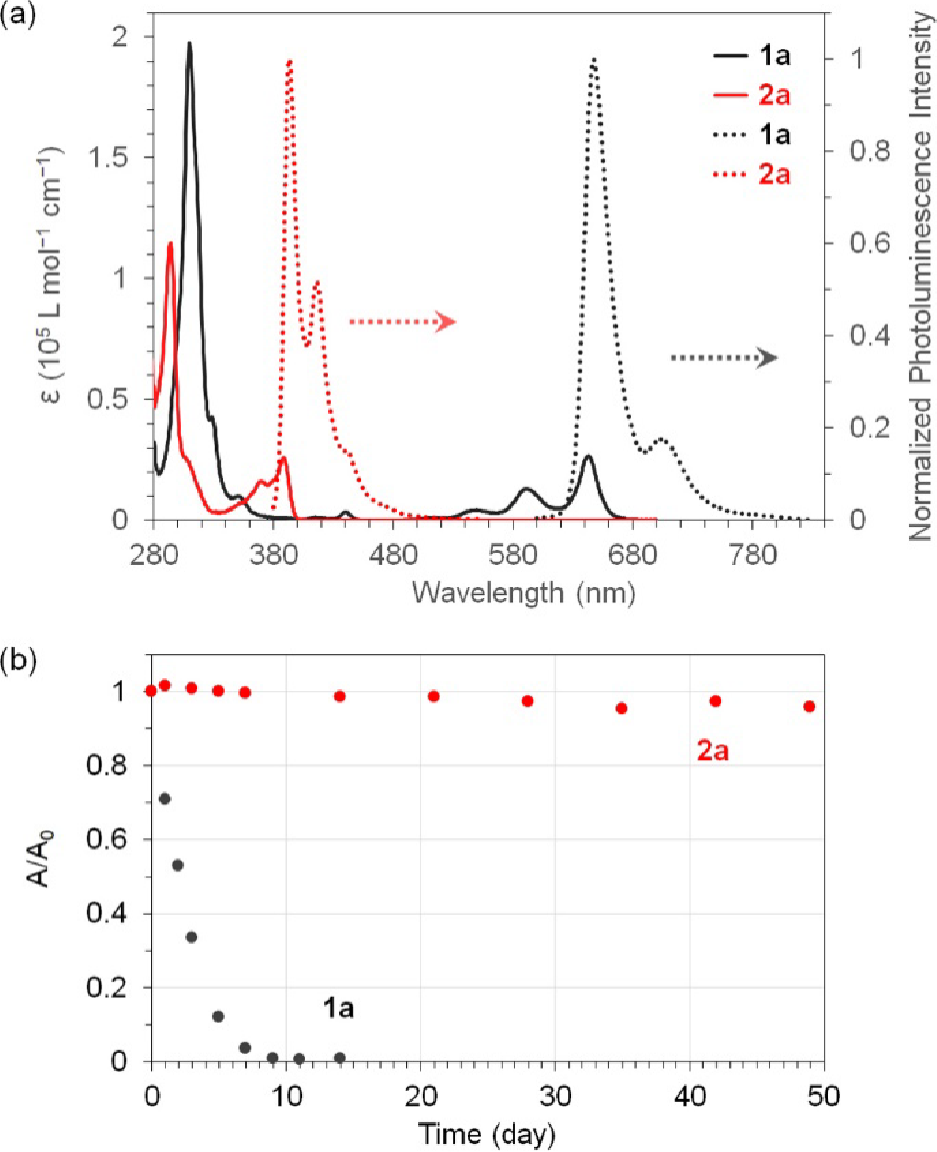
(a) UV–vis absorption (solid line) and photoluminescence (dashed line) of **1a** and **2a** in toluene. (The concentration was 10 μM for absorption and 1 μM for photoluminescence; the excitation wavelength was 590 nm for **1a** and 370 nm for **2a**.) (b) Relative absorbance as a function of time as measured from the toluene solutions of **1a** and **2a** (10 μM). (The absorbance was measured at the longest-wavelength absorption: 642 nm for **1a**, 389 nm for **2a**.)

The electronic structures and properties of compounds **1a** and **2a** were studied by using both computational and experimental methods. Figure 2b compares the frontier molecular orbitals of **1a** and **2a**, calculated at the B3LYP level of density functional theory (DFT) with a 6-311++G(d,p) basis set, highlighting the distinct phase and nodal properties of the two molecules. The HOMO of **1a** exhibits C_2h_ symmetry, while that of **2a** displays C_2v_ symmetry. The lowest unoccupied molecular orbital (LUMO) of **1a** spans the entire π-backbone with C_2v_ symmetry, whereas the LUMO of **2a**, characterized by C_2h_ symmetry, is localized on the two naphthalene moieties. The calculated HOMO energy levels of **1a** (−4.91 eV) and **2a** (−5.20 eV) differ by 0.29 eV, suggesting that both of them can function as p-type semiconductors, while the LUMO energy level of **1a** (−2.95 eV) is significantly lower than that of **2a** (−1.39 eV) by 1.56 eV. In the test window of cyclic voltammetry (CV), **1a** exhibited one reversible and one pseudoreversible oxidation wave, while **2a** showed one reversible oxidation wave. Based on the first oxidation potentials, the HOMO energy levels of **1a** and **2a** are estimated to be −5.47 and −5.64 eV, respectively. The lower HOMO energy level of **2a** is consistent with DFT calculations. Figure 3a compares the UV–vis absorption and photoluminescence spectra of **1a** and **2a** in toluene solutions. The solution of **2a** is essentially transparent to visible light, with the longest-wavelength absorption maximum at 389 nm, significantly blue-shifted by 1.26 eV relative to **1a**. This observation agrees with the DFT-calculated HOMO–LUMO gaps. The solution **2a** in toluene exhibits strong blue luminescence with a quantum yield of 53% when excited at 370 nm, while that of **1a** exhibits red luminescence with a quantum yield of 34% when excited at 590 nm.

UV–vis spectroscopy was also used to monitor the stability of **1a** and **2a** in air-saturated toluene exposed to ambient air and light. Figure 3b compares the relative absorbance of **1a** and **2a** over time under the same conditions, revealing that the characteristic absorption of **1a** disappeared after 9 days, while that of **2a** decreased by only 4.2% after 49 days. The instability of **1a** is known as a result of self-sensitized photooxidation via a Diels–Alder reaction between the diene moiety in pentacene and singlet oxygen molecule.^29,30^ Therefore, the stability of **2a** can be attributed to its transparency to visible light, which inhibits self-sensitization, and its 6,13-DHDAP backbone, which lacks a diene moiety for the Diels–Alder reaction.

**Figure 4:**
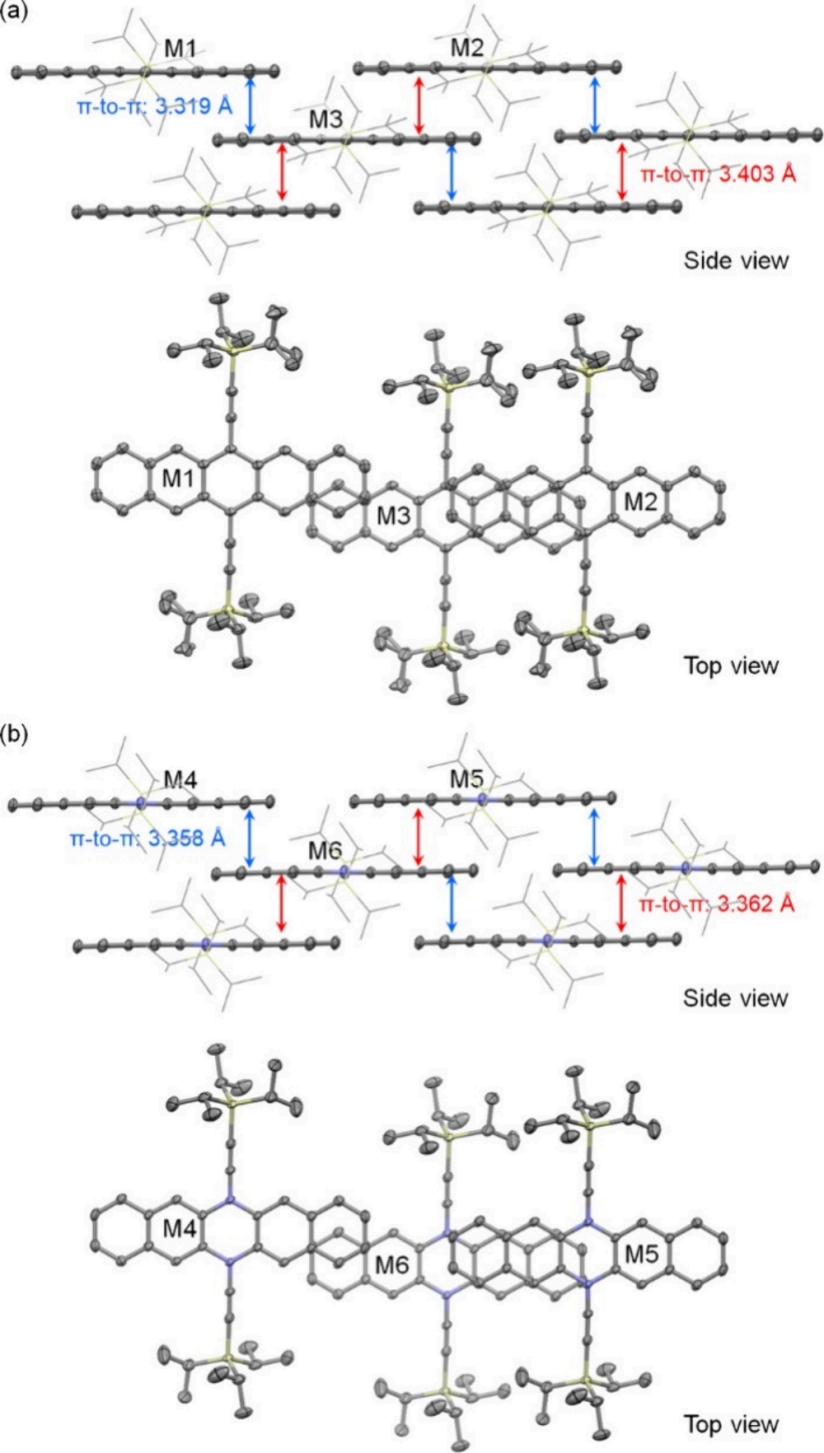
2D π-stacking of **1a** (a) and **2a** (b) in the crystals (hydrogen atoms are removed for clarity, triisopropylsilylethynyl groups are shown as wires in the side view, and other atoms are shown as ellipsoids set at 50% probability).

A key aspect of molecular orbital tuning is ensuring that the geometry and molecular packing of semiconductor molecules remain unchanged or exhibit only negligible variations when the frontier molecular orbitals are altered. However, achieving this retention of the same crystal structure is challenging and has rarely been realized in the field of organic semiconductors. The crystal structure of **1a** was reported by Anthony in 2001.^15^ To confirm that *N*,*N*′-diethynylated 6,13-DHDAP and the corresponding 6,13-diethynylated pentacene meet this criterion, single crystals of **2a** and **2b** as well as **1b** were grown by the slow diffusion of methanol vapor into their CH_2_Cl_2_ solutions and subjected to X-ray crystallography. Both **2a** and **2b** exhibit a planar diazapentacene backbone, with their acetylene moieties remaining in the same plane as the diazapentacene core. The crystal structures of **1a** and **2a** have nearly identical unit cell parameters, as summarized in Table S2 in the Supporting Information. Figure 4 compares the crystal structures of **1a** and **2a**. The side view in Figure 4a shows the 2D π–π stacking of **1a** with a brickwork arrangement, which involves two slightly different distances between the adjacent π-planes. The shorter π–π distance (3.319 Å) is associated with a small overlap between the terminal benzenoid rings, as demonstrated with molecules M1 and M3 in the top view. The longer π–π distance (3.403 Å) is associated with a larger overlap between two and a half hexagonal rings, as demonstrated with molecules M2 and M3. Figure 4b illustrates the 2D π–π stacking of **2a**, which is essentially identical to that of **1a** except for slightly different π–π distances, 3.358 and 3.362 Å.

**Figure 5:**
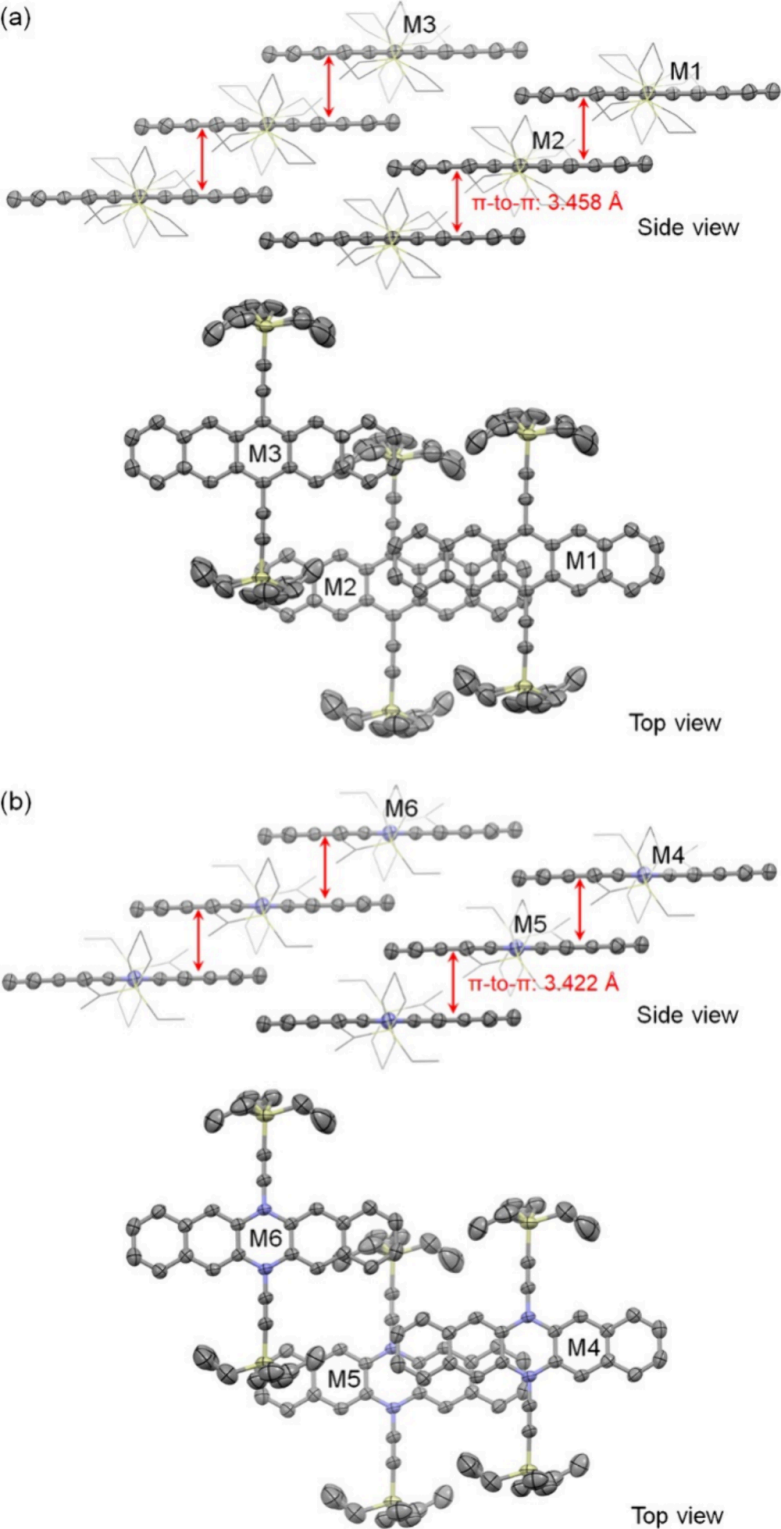
1D π-stacking of **1b** (a) and **2b** (b) in the crystals (hydrogen atoms are removed for clarity, triethylsilylethynyl groups are shown as wires in the side view, and other atoms are shown as ellipsoids set at 50% probability).

Similar to 6,13-bis(trialkylsilylethynyl)pentacene,^31^ the molecular packing of *N,N*′-ethynylated 6,13-DHDAP is controlled by the size of the trialkylsilyl group. The crystal structures of compounds **1b** and **2b**, unlike those of **1a** and **2a**, exhibit one-dimensional (1D) π–π stacking with an offset due to their smaller triethylsilyl groups compared to the triisopropyl groups in **1a** and **2a**. The side view in Figure 5a shows the 1D π–π stacking of **1b** with a π–π distance of 3.458 Å, which corresponds to an overlap between two and a half hexagonal rings, as illustrated by molecules M1 and M2 in the top view. Adjacent π-stacks of **1b** interact through the triethylsilyl group and the terminal benzenoid ring. Figure 5b illustrates the 1D π–π stacking of **2b**, which is essentially identical to that of **1b**, except for a slightly shorter π–π distance of 3.422 Å.

To calculate the charge transfer integral for holes, different dimers within the crystal structures of compounds **1a**/**b** and **2a**/**b** were identified, as illustrated in Figures 4 and 5, respectively. These dimers define the direction of the charge transfer. The distances between the molecular centroids and the hole transfer integrals for each dimer are summarized in Table 1. Additionally, the reorganization energy for holes was calculated for each molecule at the 6-311++G(d,p) level of DFT. These values, along with the hole transfer integrals, were then used to calculate the Marcus hopping rate and hole mobility, all of which are included in Table 1. The hole mobility of compound **1a** calculated in this study is comparable to the reported value of 1.49 cm^2^ V^–1^ s^–1^ obtained using DFT calculations at the B3LYP/6-31G(d,p) level.^32^ However, it is lower than the reported value of 2.25 cm^2^ V^–1^ s^–1^, which was obtained with thermal disorder taken into consideration.^33^ It is found that although compound **1a** adopts 2D π–π stacking, its hole transport is essentially one-dimensional along the direction of the π–π stacking between M2 and M3. The hole transfer integral of **1a**, and consequently its Marcus hopping rate and hole mobility between M1 and M3, is much smaller than those between M2 and M3. In contrast, the hole transport of compound **2a** is two-dimensional, with more balanced transfer integrals along two directions: 22.8 meV between M4 and M6, and 50.7 meV between M5 and M6. Both of these values are larger than the corresponding values for **1a**. As a result, the calculated hole mobility of **2a** is higher than that of **1a** in both directions, despite **2a** having a higher reorganization energy than **1a**. The enhanced hole transport in **2a** compared to that in **1a** is attributed to the different phase and nodal properties of the HOMO of **2a**, showcasing the effectiveness of molecular orbital tuning. Both **1b** and **2b** crystals exhibit one-dimensional hole transport due to their 1D π–π stacking. The hole transport between adjacent π-stacks is negligible compared to that within the π-stack. Compound **2b** has a hole transfer integral of 56.3 meV along the π–π stacking direction, which is higher than that of **1b** (43.7 meV). Consequently, the calculated mobility of **2b** is higher than that of **1b**, despite **2b** having a higher reorganization energy.

**Table 1:** Calculated Reorganization Energy, Hole Transfer Integral, Marcus Hopping Rate, and Hole Mobilities

molecule	reorganization energy (meV)	dimer	distance (Å)^a^	transfer integral (meV)^b^	Marcus hopping rate (meV)	hole mobility (cm^2^ V^–1^ s^–1^)^c^
**1a**	136.51	M1–M3	10.21	4.3	0.15	0.001
		M2–M3	7.57	32.5	8.42	1.387
**2a**	158.11	M4–M6	10.27	22.8	3.12	0.164
		M5–M6	7.45	50.7	15.45	2.093
**1b**	138.89	M1–M2	7.25	43.7	14.74	2.209
		M2–M3	10.27	3.9	0.12	0.00025
		M3–M1	13.38	6.3	0.31	0.003
**2b**	164.16	M4–M5	7.24	56.3	17.63	2.697
		M5–M6	10.14	4.0	0.09	0.00013
		M6–M4	13.34	0	0	0

a Measured between the centroids of the two molecules.

b Hole transfer integral is calculated at the 6-311++G(d,p) level of DFT.

c The computational methods are detailed in the Supporting Information.

**Figure 6:**
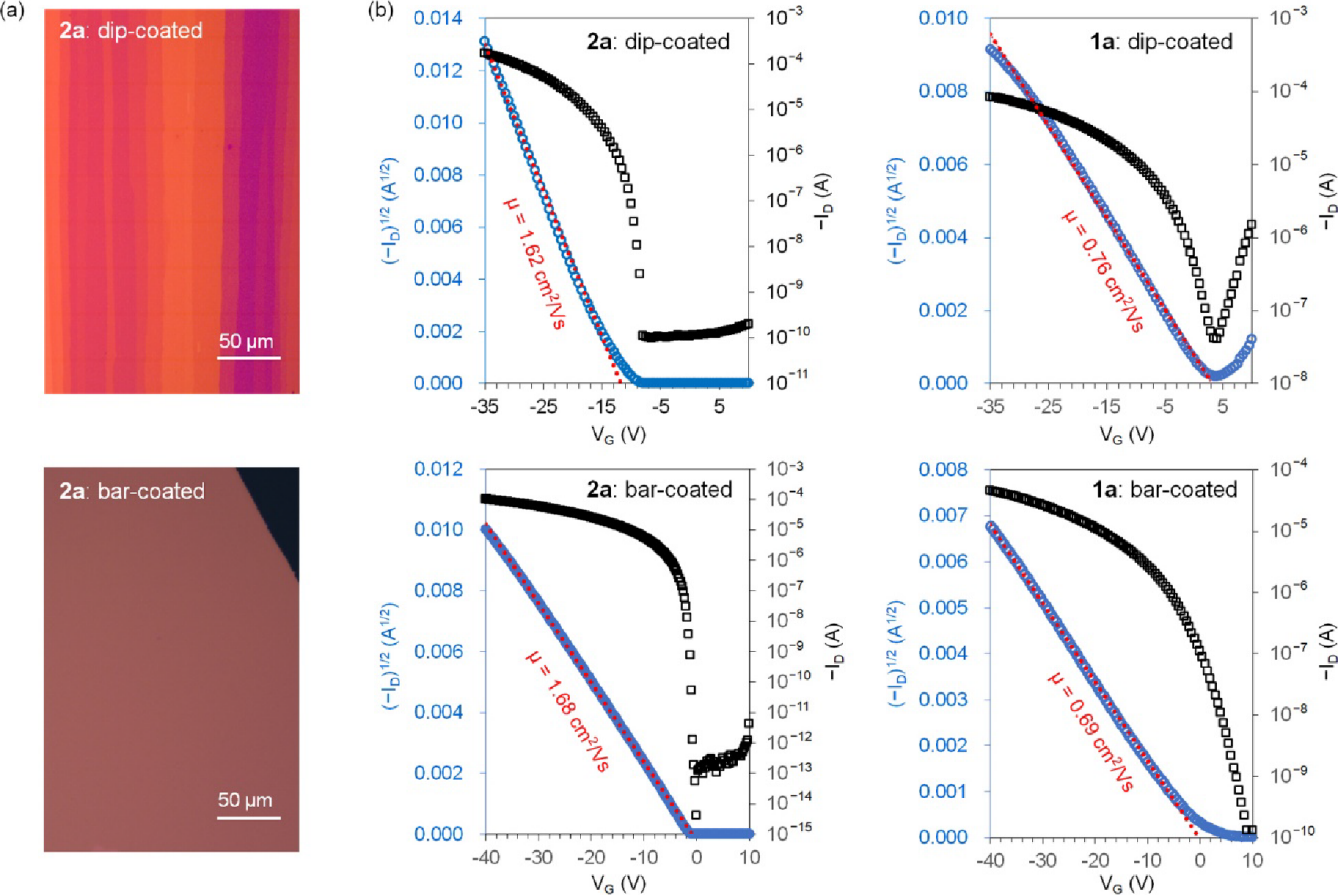
(a) Polarized light micrographs for the dip-coated and bar-coated films of **2a**. (b) Typical transfer *I–V* curves measured from the OFETs of **2a** and **1a** (the *W*/*L* is 13.8 for the dip-coated OFET of **2a**, 5.9 for the dip-coated OFET of **1a**, 8.4 for the bar-coated OFET of **2a**, and 8.3 for the bar-coated OFET of **1a**. *C*_*i*_ is 28 nF/cm^2^ for the dip-coated OFETs and 11 nF/cm^2^ for the bar-coated OFETs. The *I–V* characteristics of dip-coated OFETs were measured in ambient air, and those of bar-coated OFETs were measured in a N_2_ atmosphere).

To compare the semiconductor performance of compounds **1a/b** and **2a/b** in OFETs, two solution-based fabrication methods—dip coating and bar coating—^34−36^were employed to fabricate bottom-gate top-contact devices. For dip coating, films of these compounds were prepared on a silicon substrate layered with thermally grown silica, solution-processed alumina,^37^ and either 12-cyclohexyldodecylphosphonic acid (CDPA)^38^ or 12-methoxydodecylphosphonic acid (MODPA)^39^ as composite dielectric materials. The top-contact electrodes were formed by the vacuum deposition of gold through a shadow mask so that the resulting conduction channel was roughly parallel to the film growth direction. The solvent, pulling speed, and solution concentration used in the dip-coating process were optimized, resulting in films of **1a** and **2a** composed of aligned microribbons or fibrous crystallites (Figure 6a and Figure S13). However, the dip-coated films of **1b** and **2b** consisted of narrower microribbons or fibrous crystallites with poorer alignment, as observed in the polarized light micrographs (Figure S13) and AFM images (Figure S15). For bar coating,^40^ solutions of the four compounds were applied to SiO_2_/Si substrates coated with a polymer of divinyltetramethyldisiloxane bis(benzocyclobutene) (BCB). The solvent, solution concentration, substrate temperature, and substrate moving speed used in the bar-coating process were optimized, resulting in thin films of **1a** and **2a** with millimeter-sized domains, full coverage, and uniform thickness (Figure 6a and Figure S14). However, the bar-coated films of **1b** and **2b** were composed of microribbons with varied thickness, as observed in the polarized light micrographs (Figure S14) and AFM images (Figure S16).

The molecular arrangement and morphology of these films were analyzed using X-ray diffraction (XRD), grazing incidence wide-angle X-ray scattering (GIWAXS),^41−43^ and atomic force microscopy (AFM). The out-of-plane X-ray diffraction patterns for dip-coated and bar-coated films of **1a** and **2a** (Figure S17 and S18) all showed three peaks corresponding to the (001), (002), and (003) diffractions derived from the single-crystal structures. Meanwhile, the diffraction patterns for films of **1b** and **2b** showed only (001) and (002) peaks, suggesting lower crystallinity in these films compared to those of **1a** and **2a**. The GIWAXS patterns of the dip-coated and bar-coated films of **1a/b** and **2a/b** all exhibited (001) diffraction along the q_*z*_ (out-of-plane) axis, consistent with the out-of-plane X-ray diffraction patterns. This indicates that the (001) crystal plane is parallel to the substrate surface, and thus, the π-backbones adopt an edge-on orientation on the substrate surface. The π-planes of **1a** and **2a** form angles of 69.6 and 70.4°, respectively, with the substrate surface, while those of **1b** and **2b** form angles of 52.4 and 53.2°, respectively, with the substrate surface (Figure S19). When the incident X-ray beam was perpendicular to the film growth direction of **1a** and **2a**, the GIWAXS patterns exhibited (10*l*) diffractions and higher-order (20*l*) diffractions, similar to the reported GIWAXS patterns of blade-coated films of compound **1a**.^44^ This suggests that the film growth direction is roughly along the *a-*axis of the crystal unit cell (Figures S21 and S22). The GIWAXS patterns of the dip-coated films of **1b** and **2b**, unlike those of **1a** and **2a**, exhibited small arcs, indicative of less oriented domains, where the crystallites are oriented over a range of angles. On the other hand, the GIWAXS patterns of the bar-coated films of **1b** and **2b** exhibited dots corresponding to (01*l*) diffractions when the incident X-ray beam was parallel to the film growth direction. This suggests that the major film growth direction is roughly along the *a-*axis of the crystal unit cell (Figures S24 and S26).

Typical AFM images of the dip-coated films of **1a** and **2a** (Figure S15) revealed flat surfaces of the microribbons, with section analysis showing steps of 4 to 6 nm between adjacent microribbons, corresponding to two to three molecular layers. Meanwhile, typical AFM images of the bar-coated film of **1a** and **2a** (Figure S16) revealed uniform thicknesses of 12 and 24 nm, respectively. These thicknesses correspond to about seven molecular layers for **1a** and 14 molecular layers for **2a**. On the other hand, AFM images of the dip-coated films of **1b** and **2b** (Figure S15) showed deep and wide gaps between microribbons, while those of the bar-coated films of **1b** and **2b** (Figure S16) exhibited rougher surfaces compared to those of **1a** and **2a**.

Figure 6 presents the typical transfer *I*–*V* curves for OFETs fabricated from compounds **1a** and **2a** using two different methods. The field-effect mobility for holes in the saturated regime was extracted using the equation *I*_DS_ = (μ*WC*_*i*_/2*L*)(*V*_GS_–*V*_T_),^2^ where *I*_DS_ is the drain current, μ is the field-effect mobility, *C*_*i*_ is the capacitance per unit area for the corresponding dielectric, *W* is the channel width, *L* is the channel length, and *V*_GS_ and *V*_T_ are the gate and threshold voltages, respectively. As summarized in Table 2, the average field-effect mobility of **1a** (0.72 ± 0.13 cm^2^ V^–1^ s^–1^) is comparable to the values reported for drop-cast films of **1a** (0.65 ± 0.35 and 0.73 ± 0.06 cm^2^ V^–1^ s^–1^),^45,46^ although it is lower than those observed in single-crystal arrays (1.54 ± 0.26 cm^2^ V^–1^ s^–1^).^47^ However, the highest field-effect mobility of **1a** so far obtained from lattice-strained single crystalline films (8.1 ± 1.2 cm^2^ V^–1^ s^–1^) may be overestimated, as it was extracted from transfer *I*–*V* curves exhibiting apparent double-slope nonideality.^48^ The average field-effect mobilities of **2a** in both dip-coated and bar-coated films exceed 1 cm^2^ V^–1^ s^–1^. The bar-coated films, in particular, exhibited a higher mobility, reaching up to 1.98 cm^2^ V^–1^ s^–1^, which is close to the calculated value (2.09 cm^2^ V^–1^ s^–1^). The on/off ratio is >1 × 10^6^ for the dip-coated OFETs of **2a**, and >1 × 10^9^ for the bar-coated OFETs of **2a**. Similarly, the bar-coated OFETs of **1a** showed higher mobility compared to that of the dip-coated OFETs of the same material. This increase in mobility is attributed to the single-crystal nature of the large-sized domains in the bar-coated films. The mobilities of **2a** are more than double those of **1a**, aligning qualitatively with the calculated values (Table 1). Since the thin films of **1a** and **2a** fabricated using the same method exhibit similar crystallinity and morphology, the significantly enhanced mobility of **2a** compared to **1a** should be attributed to the molecular structure itself, confirming the effectiveness of molecular orbital tuning.

**Table 2:** Hole Mobilities for OFETs of **1a/b** and **2a/b**

molecules	fabrication method	μ (cm^2^ V^–1^ s^–1^)^a^
**1a**	dip coating	0.55 ± 0.13
		highest: 0.76
	bar coating	0.72 ± 0.13
		highest: 0.94
**2a**	dip coating	1.29 ± 0.20
		highest: 1.62
	bar coating	1.64 ± 0.17
		highest: 1.98
**1b**	dip coating	(5.8 ± 3.8) × 10^–3^
		highest: 1.46 × 10^–2^
	bar coating	0.21 ± 0.06
		highest: 0.34
**2b**	dip coating	(6.0 ± 4.3) × 10^–3^
		highest: 1.62 × 10^–2^
	bar coating	0.43 ± 0.09
		highest: 0.57

a The average mobilities were measured from 47 independent dip-coated OFETs for **1a**, 70 for **2a**, 28 for **1b**, and 30 for **2b** in ambient air, and from 16 independent bar-coated OFETs for each compound of **1a/b** and **2a/b** in a N_2_ atmosphere.

In comparison to **1a** and **2a**, the triethylsilyl-substituted molecules **1b** and **2b** in dip-coated and bar-coated films exhibited lower field-effect mobilities, as shown in Table 2. This reduction in mobility for **1b** and **2b** can be attributed to the films consisting of ribbon or fiber-like domains with lower ordering and poorer orientation than those of **1a** and **2a**, as observed from the polarized light micrographs, XRD, GIWAXS, and AFM images. The charge transport in the dip-coated films of **1b** and **2b** was hindered by deep and wide grain boundaries between these microribbons or fibrous crystallites, resulting in much lower measured mobilities than the intrinsic values predicted by theoretical calculations. Compared to the dip-coated films, the bar-coated films of **1b** and **2b** exhibited mobilities higher by 1 order of magnitude, closer to the intrinsic values predicted by theoretical calculations. In the bar-coated films with similar crystallinity and morphology, the mobility of **2b** approximately doubles that of **1b**, qualitatively aligning with the theoretically calculated values and thereby supporting the effectiveness of molecular orbital tuning.

## Conclusions

In conclusion, this study demonstrates the concept of molecular orbital tuning of organic semiconductors using *N,N*′-diethynylated 6,13-DHDAPs (**2a** and **2b**). The two new molecules retain the same molecular geometry and π–π stacking as the parent pentacene derivatives (**1a** and **1b**), as revealed by X-ray crystallography, but they alter the frontier molecular orbitals in terms of phase, nodal properties, and energy levels. Theoretical calculations based on the crystal structures suggest that **2a** and **2b** have the potential to improve the hole mobilities of the parent compounds (**1a** and **1b**) by enhancing the hole transfer integral. This prediction was supported by the OFETs fabricated using dip- and bar-coating methods. Both types of the devices for **2a** exhibited hole mobility exceeding 1 cm^2^ V^–1^ s^–1^, more than twice that of the respective devices for **1a**. The field-effect mobility of **2b** in bar-coated OFETs also doubled that of **1b**, although the mobilities of **1b** and **2b** are both lower than those of **1a** and **2a** due to their films having lower ordering and poorer orientation. Additionally, unlike its pentacene parent, **2a** is not only transparent to visible light but also exhibits significantly enhanced environmental stability toward light and air, making it more promising for wider application in organic electronic devices.

Molecular orbital tuning, which can alternatively be termed molecular orbital engineering, involves designing and producing frontier molecular orbitals for organic semiconductors. Specifically, it modifies the frontier molecular orbitals of an organic semiconductor without altering its shape and molecular packing in the crystal structure by substituting atoms in its π-backbone. This modification changes the charge transfer integral, leading to an increase or decrease in the rate of charge transport in organic semiconductors. While such modifications do not always guarantee an enhanced charge transport rate, the concept of molecular orbital tuning provides a novel strategy for designing new organic semiconductors. It allows for the prediction of transfer integral and charge carrier mobility before the synthesis of new molecules based on the crystal structure of known organic semiconductors. If the predicted charge transfer integral and charge carrier mobility are higher than those of the known organic semiconductor, the new molecule becomes a promising candidate for improved semiconductor performance. Research on new organic semiconductors designed using this strategy is in progress in our laboratory.

## Supporting Information Available

Details of synthesis, characterization of new compounds, X-ray crystallography, DFT calculations, and fabrication and characterization of thin films and field-effect transistors (PDF)

## References

[ref1] Brédas J. L.; Calbert J. P.; da Silva Filho D. A.; Cornil J. Organic semiconductors: A theoretical characterization of the basic parameters governing charge transport. Natl. Acad. Sci. U.S.A. 2002, 99, 5804–5809. 10.1073/pnas.092143399. PMC122857 11972059

[ref2] Marcus R. A. On the Theory of Oxidation-Reduction Reactions Involving Electron Transfer. I. J. Chem. Phys. 1956, 24, 966–978. 10.1063/1.1742723.

[ref3] Marcus R. A. Electron transfer reactions in chemistry. Theory and experiment. Protein electron transfer, Rev. Mod. Phys. 1993, 65, 599–610. 10.1103/RevModPhys.65.599.

[ref4] Shan B.; Miao Q. Molecular design of n-type organic semiconductors for high-performance thin film transistors. Tetrahedron Lett. 2017, 58, 1903–1911. 10.1016/j.tetlet.2017.04.023.

[ref5] Someya T.; Bao Z.; Malliaras G. G. The rise of plastic bioelectronics. Nature 2016, 540, 379–385. 10.1038/nature21004. 27974769

[ref6] Liu Z.; Zhang C.; Xiang L.; Zhang F.; Di C. Organic transistors-driven wearable electronics for smart life. Wearable Electron. 2024, 1, 211–227. 10.1016/j.wees.2024.09.004.

[ref7] Wang C.; Liu Y.; Guo Y. Intrinsically flexible organic phototransistors for bioinspired neuromorphic sensory system. Wearable Electron. 2024, 1, 41–52. 10.1016/j.wees.2024.05.001.

[ref8] Brédas J.-L.; Beljonne D.; Coropceanu V.; Cornil J. Charge-transfer and energy-transfer processes in π-conjugated oligomers and polymers: a molecular picture. Chem. Rev. 2004, 104, 4971–5004. 10.1021/cr040084k. 15535639

[ref9] Mas-Torrent M.; Rovira C. Role of molecular order and solid-state structure in organic field-effect transistors. Chem. Rev. 2011, 111, 4833–4856. 10.1021/cr100142w. 21417271

[ref10] Dong H.; Fu X.; Liu J.; Wang Z.; Hu W. 25th Anniversary Article: Key Points for High-Mobility Organic Field-Effect Transistors. Adv. Mater. 2013, 25, 6158–6183. 10.1002/adma.201302514. 24105677

[ref11] Yao Z.-F.; Wang J.-Y.; Pei J. Control of π–π stacking via crystal engineering in organic conjugated small molecule crystals. Cryst. Growth Des. 2018, 18, 7–15. 10.1021/acs.cgd.7b01385.

[ref12] Guo J.; Shi C.; Zhen Y.; Hu W. Rational Control of Packing Arrangements in Organic Semiconducting Materials toward High-Performance Optoelectronics. Acc. Mater. Res. 2024, 5, 907–919. 10.1021/accountsmr.4c00054.

[ref13] Zhang Y.; Wang Y.; Gao C.; Ni Z.; Zhang X.; Hu W.; Dong H. Recent advances in n-type and ambipolar organic semiconductors and their multi-functional applications. Chem. Soc. Rev. 2023, 52, 1331–1381. 10.1039/D2CS00720G. 36723084

[ref14] Yu P.; Zhen Y.; Dong H.; Hu W. Crystal engineering of organic optoelectronic materials. Chem. 2019, 5, 2814–2853. 10.1016/j.chempr.2019.08.019.

[ref15] Anthony J. E.; Brooks J. S.; Eaton D. L.; Parkin S. R. Functionalized Pentacene: Improved Electronic Properties from Control of Solid-State Order. J. Am. Chem. Soc. 2001, 123, 9482–9483. 10.1021/ja0162459. 11562247

[ref16] aPayne M. M.; Parkin S. R.; Anthony J. E.; Kuo C.-C.; Jackson T. N. Organic Field-Effect Transistors from Solution-Deposited Functionalized Acenes with Mobilities as High as 1 cm^2^/V·s. J. Am. Chem. Soc. 2005, 127, 4986–4987. 10.1021/ja042353u. 15810810

[ref17] Miao Q.; Nguyen T.-Q.; Someya T.; Blanchet G. B.; Nuckolls C. Synthesis, assembly, and thin film transistors of dihydrodiazapentacene: an isostructural motif for pentacene. J. Am. Chem. Soc. 2003, 125, 10284–10287. 10.1021/ja036466+. 12926952

[ref18] Tang Q.; Zhang D.; Wang S.; Ke N.; Xu J.; Yu J. C.; Miao Q. A Meaningful Analogue of Pentacene: Charge Transport, Polymorphs, and Electronic Structures of Dihydrodiazapentacene. Chem. Mater. 2009, 21, 1400–1405. 10.1021/cm9001916.

[ref19] Xie G.; Hauschild M.; Hoffmann H.; Ahrens L.; Rominger F.; Borkowski M.; Marszalek T.; Freudenberg J.; Kivala M.; Bunz U. H. F. 5,7,12,14-Tetrafunctionalized 6,13-Diazapentacenes. Chem.—Eur. J. 2020, 26, 799–803. 10.1002/chem.201904516. 31609025 PMC7004126

[ref20] Xie G.; Bojanowski N. M.; Brosius V.; Wiesner T.; Rominger F.; Freudenberg J.; Bunz U. H. F. Stable N,N’-Diarylated Dihydrodiazaacene Radical Cations. Chem.—Eur. J. 2021, 27, 1976–1980. 10.1002/chem.202004548. 33226146 PMC7898594

[ref21] Gu X.; Shan B.; He Z.; Miao Q. N-Phenylated N-Heteroacenes: Synthesis, Structures, and Properties. ChemPlusChem. 2017, 82, 1034–1038. 10.1002/cplu.201600465. 31961616

[ref22] Liang Z.; Tang Q.; Xu J.; Miao Q. Soluble and stable N-heteropentacenes with high field-effect mobility. Adv. Mater. 2011, 23, 1535–1539. 10.1002/adma.201004325. 21449057

[ref23] Miao Q. Ten Years of N-Heteropentacenes as Semiconductors for Organic Thin-Film Transistors. Adv. Mater. 2014, 26, 5541–5549. 10.1002/adma.201305497. 24585514

[ref24] Miao S.; Appleton A. L.; Berger N.; Barlow S.; Marder S. R.; Hardcastle K. I.; Bunz U. H. F. 6,13-Diethynyl-5,7,12,14-tetraazapentacene. Chem.—Eur. J. 2009, 15, 4990–4993. 10.1002/chem.200900324. 19338039

[ref25] Liu Y.-Y.; Song C.-L.; Zeng W.-J.; Zhou K.-G.; Shi Z.-F.; Ma C.-B.; Yang F.; Zhang H.-L.; Gong X. High and Balanced Hole and Electron Mobilities from Ambipolar Thin-Film Transistors Based on Nitrogen-Containing Oligoacences. J. Am. Chem. Soc. 2010, 132, 16349–16351. 10.1021/ja107046s. 20979424

[ref26] Zhang Z.; Wang Z.; Aratani N.; Zhu X.; Zhang Q. Seeing Is Believing: A Wavy N-Heteroarene with 20 Six-Membered Rings Linearly Annulated in a Row. CCS Chem. 2022, 4, 3491–3496. 10.31635/ccschem.022.202202013.

[ref27] Dubey R. K.; Melle-Franco M.; Mateo-Alonso A. Twisted Molecular Nanoribbons with up to 53 Linearly-Fused Rings. J. Am. Chem. Soc. 2021, 143, 6593–6600. 10.1021/jacs.1c01849. 33876941

[ref28] Mansfield S. J.; Campbell C. D.; Jones M. W.; Anderson E. A. A robust and modular synthesis of ynamides. Chem. Commun. 2015, 51, 3316–3319. 10.1039/C4CC07876D. 25374291

[ref29] Maliakal A.; Raghavachari K.; Katz H.; Chandross E.; Siegrist T. Photochemical Stability of Pentacene and a Substituted Pentacene in Solution and in Thin Films. Chem. Mater. 2004, 16, 4980–4986. 10.1021/cm049060k.

[ref30] Kaur I.; Jia W.; Kopreski R. P.; Selvarasah S.; Dokmeci M. R.; Pramanik C.; McGruer N. E.; Miller G. P. Substituent Effects in pentacenes: Gaining Control over HOMO–LUMO Gaps and Photooxidative Resistances. J. Am. Chem. Soc. 2008, 130, 16274–16286. 10.1021/ja804515y. 19006312

[ref31] Anthony J. E.; Eaton D. L.; Parkin S. R. A Road Map to Stable, Soluble, Easily Crystallized Pentacene Derivatives. Org. Lett. 2002, 4, 15–18. 10.1021/ol0167356. 11772079

[ref32] Li C.-H.; Huang C.-H.; Kuo M.-Y. Halogenated 6,13-bis(triisopropylsilylethynyl)-5,7,12,14-tetraazapentacene: applications for ambipolar air-stable organic field-effect transistors. Phys. Chem. Chem. Phys. 2011, 13, 11148–11155. 10.1039/c1cp20391f. 21566822

[ref33] Zhang N.-X.; Ren A.-M.; Ji L.-F.; Zhang S.-F.; Guo J.-F. Theoretical Investigations on Molecular Packing Motifs and Charge Transport Properties of a Family of Trialkylsilylethynyl-Modified pentacenes/Anthradithiophenes. J. Phys. Chem. C 2018, 122, 18880–18894. 10.1021/acs.jpcc.8b06527.

[ref34] Zhang Z.; Peng B.; Ji X.; Pei K.; Chan P. K. L. Marangoni-Effect-Assisted Bar-Coating Method for High-Quality Organic Crystals with Compressive and Tensile Strains. Adv. Funct. Mater. 2017, 27, 1703443 10.1002/adfm.201703443.

[ref35] Bai J.; Jiang Y.; Wang Z.; Sui Y.; Deng Y.; Han Y.; Geng Y. Bar-Coated Organic Thin-Film Transistors with Reliable Electron Mobility Approaching 10 cm^2^ V^–1^ s^–1^. Adv. Electron. Mater. 2020, 6, 1901002 10.1002/aelm.201901002.

[ref36] Lee S. B.; Lee S.; Kim D. G.; Kim S. H.; Kang B.; Cho K. Solutal-Marangoni-Flow-Mediated Growth of Patterned Highly Crystalline Organic Semiconductor Thin Film Via Gap-Controlled Bar Coating. Adv. Funct. Mater. 2021, 31, 2100196 10.1002/adfm.202100196.

[ref37] Chu M.; Fan J.-X.; Yang S.; Liu D.; Ng C. F.; Dong H.; Ren A.-M.; Miao Q. Halogenated Tetraazapentacenes with Electron Mobility as High as 27.8 cm^2^ V^–1^ s^–1^ in Solution-Processed n-Channel Organic Thin-Film Transistors. Adv. Mater. 2018, 30, 1803467 10.1002/adma.201803467. 30066472

[ref38] Liu D.; He Z.; Su Y.; Diao Y.; Mannsfeld S. C. B.; Bao Z.; Xu J.; Miao Q. Self-Assembled Monolayers of Cyclohexyl-Terminated Phosphonic Acids as a General Dielectric Surface for High-Performance Organic Thin-Film Transistors. Adv. Mater. 2014, 26, 7190–7196. 10.1002/adma.201402822. 25205623

[ref39] Liu D.; Xu X.; Su Y.; He Z.; Xu J.; Miao Q. Self-Assembled Monolayers of Phosphonic Acids with Enhanced Surface Energy for High-Performance Solution-Processed N-Channel Organic Thin-Film Transistors. Angew. Chem., Int. Ed. 2013, 52, 6222–6227. 10.1002/anie.201300353. 23650029

[ref40] Zhao Y.; Sheng Q.; Ke S.; Wu R.; He L.; Ren X.; Peng B.; Li H. Direct Solution Deposition of Large-Area Non-Solvated Fullerene Single-Crystal Films for High-Performance n-Type Field-Effect Transistors. Small 2024, 20, 2404770 10.1002/smll.202404770. 39105335

[ref41] Steele J. A.; Solano E.; Hardy D.; Dayton D.; Ladd D.; White K.; Chen P.; Hou J.; Huang H.; Saha R. A.; Wang L.; Gao F.; Hofkens J.; Roeffaers M. B. J.; Chernyshov D.; Toney M. F. How to GIWAXS: Grazing Incidence Wide Angle X-RayScattering Applied to Metal Halide Perovskite Thin Films. Adv. Energy Mater. 2023, 13, 2300760 10.1002/aenm.202300760.

[ref42] Zhang Y.; Liu Q.; Gao C.; Xie Z.; Hu B.; Dong H. Packing Adjustment Towards High Mobility Luminescent Conjugated Polymers. Chem. Res. Chin. Univ. 2023, 39, 731–735. 10.1007/s40242-023-3180-4.

[ref43] Zhang Y.; Xu C.; Wang P.; Gao C.; Li W.; Ni Z.; Han Y.; Zhao Y.; Geng Y.; Wang Z.; Hu W.; Dong H. Universal Design and Efficient Synthesis for High Ambipolar Mobility Emissive Conjugated Polymers. Angew. Chem., Int. Ed. 2024, 63, e202319997 10.1002/anie.202319997. 38499464

[ref44] Kim K.; Nam K.; Li X.; Lee D. Y.; Kim S. H. Programmed Design of Highly Crystalline Organic Semiconductor Patterns with Uniaxial Alignment via Blade Coating for High-Performance Organic Field-Effect Transistors. ACS Appl. Mater. Interfaces 2019, 11, 42403–42411. 10.1021/acsami.9b12765. 31617995

[ref45] Chae G. J.; Jeong S.-H.; Baek J. H.; Walker B.; Song C. K.; Seo J. H. Improved performance in TIPS-pentacene field effect transistors using solvent additives. J. Mater. Chem. C 2013, 1, 4216–4221. 10.1039/c3tc30506f.

[ref46] Park S. K.; Jackson T. N.; Anthony J. E.; Mourey D. A. High mobility solution processed 6,13-bis(triisopropyl-silylethynyl) pentacene organic thin film transistors. Appl. Phys. Lett. 2007, 91, 063514 10.1063/1.2768934.

[ref47] Wang S.; Zhou S.; Tong Y.; Song Z.; Wang H.; Tang Q.; Zhao X.; Liu Y. Dielectric Selection for Solution-Processed High-Mobility TIPS-Pentacene Microwire Field-Effect Transistors. Adv. Mater. Interfaces 2019, 6, 1801984 10.1002/admi.201801984.

[ref48] Diao Y.; Tee B. C. K.; Giri G.; Xu J.; Kim D. H.; Becerril H. A.; Stoltenberg R. M.; Lee T. H.; Xue G.; Mannsfeld S. C. B.; Bao Z. Solution coating of large-area organic semiconductor thin films with aligned single-crystalline domains. Nat. Mater. 2013, 12, 665–671. 10.1038/nmat3650. 23727951

