# Supplementary material for: Molecular Orbital Tuning of Pentacene-Based Organic Semiconductors through N-Ethynylation of Dihydrodiazapentacene
Source: J Am Chem Soc. 2025 Jan 21;147(4):3459–67. doi: 10.1021/jacs.4c14775 (PMC11783513; doi:10.1021/jacs.4c14775)
Supplement: Supplementary file 1 — ja4c14775_si_001.pdf [file ja4c14775_si_001.pdf]

Supporting Information for

Molecular Orbital Tuning of Pentacene-Based Organic Semiconductors through *N*-Ethynylation of Dihydrodiazapentacene

Li Zhang,[a, b] Yujie Zhao,[d, e, f] Jiasheng Li,[g] Yuang Fu,[c] Boyu Peng,*[, d, e, f]
Jun Yang,[g, h] Xinhui Lu,[c] Qian Miao*[, a, b]

[a] *Department of Chemistry,* [b] *State Key Laboratory of Synthetic Chemistry, and* [c] *Department of Physics, The Chinese University of Hong Kong, Shatin, New Territories, Hong Kong, China*

[d] *MOE Key Laboratory of Macromolecular Synthesis and Functionalization,* [e] *International Research Center for X Polymers, and* [f] *Department of Polymer Science and Engineering, Zhejiang University, Hangzhou 310027, China*

[g] *Department of Chemistry and* [h] *State Key Laboratory of Synthetic Chemistry, The University of Hong Kong, Hong Kong, China*

* Email of the corresponding authors: pengboyu@zju.edu.cn, miaoqian@cuhk.edu.hk

Table of Contents

1. Synthesis
2. Photophysical properties
3. Cyclic Voltammetry
4. X-ray crystallography
5. Computational studies
6. Fabrication of organic field effect transistors (OFETs)
7. Characterization of thin films and OFETs
8. References

1. Synthesis

General: The reagents and starting materials employed were commercially available and used without any further purification or prepared following reported methods as indicated. Anhydrous THF was purified by an Advanced Technology Pure-Solv PS-MD-4 system. NMR spectra were recorded on a Brucker 400 MHz or 500 MHz spectrometer. Chemical shift values (δ) are expressed in parts per million using residual solvent protons as internal standard (^{1}H NMR, δ(H) is 5.32 ppm for CD_2Cl_2; ^{13}C NMR, δ(C) is 53.84 ppm for CD_2Cl_2). Mass spectra were recorded on a Q Exactive Focus Orbitrap mass spectrometer. Melting points without correction, were measured using a Nikon Polarized Light Microscope ECLIPSE 50i POL equipped with an INTEC HCS302 heating stage.

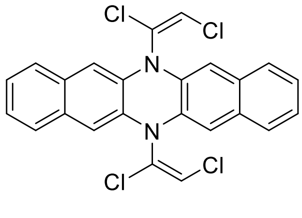

3

N,N′-bis(1,2-dichloroethenyl)-6,13-dihydro-6,13-diazapentacene (**3**)

Under a N_2 atmosphere, 176 mg (4.4 mmol, 4.4 eq.) of NaH (60% in mineral oil) was added into a solution of 6,13-dihydro-6,13-diazapentacene [1] (282 mg, 1 mmol) in 10 mL of anhydrous DMF. The mixture was stirred at room temperature for 2 hours. Then trichloroethylene (225 μL, 2.5 mmol, 2.5 eq.) was added into the mixture via a syringe and the mixture was stirred for another 24 hours. Slow addition of H_2O to the reaction mixture resulted in precipitation of crude product, which was collected through filtration and purified by silica gel column (eluent: CH_2Cl_2/hexane=1/10 to CH_2Cl_2) to afford compound **3** (345 mg, 0.73 mmol, 73%) as off-white solid. Its crystals suitable for X-ray diffraction were grown by slow diffusion of hexane to its CH_2Cl_2 solution. mp: 281-284 ^{o}C; ^{1}H NMR (400 MHz, CD_2Cl_2, δ): 7.62 (dd, *J* = 5.9, 3.3 Hz, 4H), 7.30 (dd, *J* = 6.1, 3.2 Hz, 4H), 7.05 (s, 4H), 6.97 (s, 2H); ^{13}C NMR (101 MHz, CD_2Cl_2, δ): 131.14, 129.78, 128.57, 126.86, 125.62, 122.68, 110.33; HRMS (APCI) *m*/*z*: $[M+H]^+$ calcd for $C_{24}H_{15}Cl_4N_2^+$, 472.99568; found, 472.99498.

N,N′-bis(triisopropylsilylethynyl)-6,13-dihydro-6,13-diazapentacene (**2a**)
and *N,N′*-bis(triethylsilylethynyl)-6,13-dihydro-6,13-diazapentacene (**2b**)

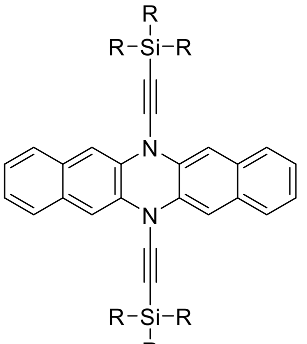

2a, R = ^{i}Pr
2b, R = Et

Under a N_2 atmosphere, compound **3** (0.2 mmol, 94 mg) was added into 20 mL of anhydrous THF. The mixture was cooled to −40 ^{o}C using a cooling bath of acetonitrile and liquid nitrogen. Then 0.8 mL (1.6 mmol, 8 eq.) of ^{n}BuLi (2.0 M in cyclohexane solution) was added into the mixture slowly at the same temperature. The reaction mixture was warmed to room temperature and stirred for 30 minutes, and then cooled to −40 ^{o}C again. Triisopropylchlorosilane (171 μL, 0.8 mmol, 4 eq.) or triethylchlorosilane (128 μL, 0.8 mmol, 4 eq.) was added into the mixture via a syringe. The resulting mixture was warmed to room temperature and stirred for 3 hours. The mixture was treated with an aquoues solution of NH_4Cl and extracted with CH_2Cl_2 for three times. The CH_2Cl_2 solution was dried with $MgSO_4$ and concentrated under a reduced presure. The crude product was puried using silica gel column chromatography (eluent: hexane) to give compound **2a** or **2b** as white crystalline solid.

2a (78.9 mg, 61%): mp. 213-215 ^{o}C; ^{1}H NMR (500 MHz, CD_2Cl_2, δ): 7.68 (s, 4H), 7.62 (dd, *J* = 6.1, 3.3 Hz, 4H), 7.32 (dd, *J* = 6.2, 3.2 Hz, 4H), 1.28 (s, 36H), 1.27 (s, 6H); ^{13}C NMR (126 MHz, CD_2Cl_2, δ): 131.29, 129.34, 126.96, 125.73, 112.11, 93.83, 77.26, 19.04, 11.98; HRMS (APCI) *m*/*z*: $[M+H]^+$ calcd for $C_{42}H_{55}N_2Si_2^+$: 643.38983; found: 643.38943.

2b (59.6 mg, 53%): mp. 203-205 ^{o}C; ^{1}H NMR (400 MHz, CD_2Cl_2, δ): 7.67 – 7.62 (m, 8H), 7.33 (dd, *J* = 6.2, 3.2 Hz, 4H), 1.19 (t, *J* = 7.9 Hz, 18H), 0.85 (q, *J* = 7.9 Hz, 12H); ^{13}C NMR (101 MHz,

CD_2Cl_2, δ): 131.29, 129.19, 126.97, 125.74, 112.03, 93.22, 78.53, 7.94, 5.14; HRMS (APCI) *m*/*z*: $[M+H]^+$ calcd for $C_{36}H_{43}N_2Si_2^+$, 559.29593; found, 559.29584.

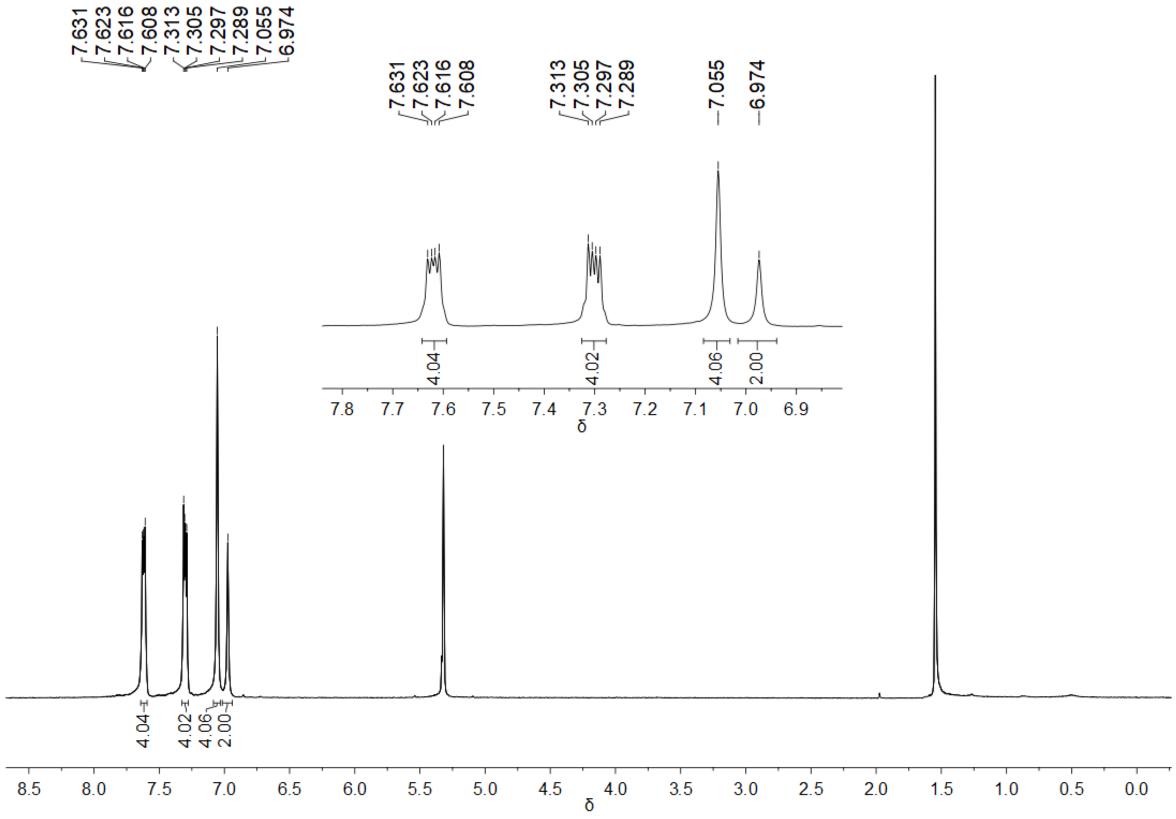

Figure S1. ^{1}H NMR spectrum of compound **3**

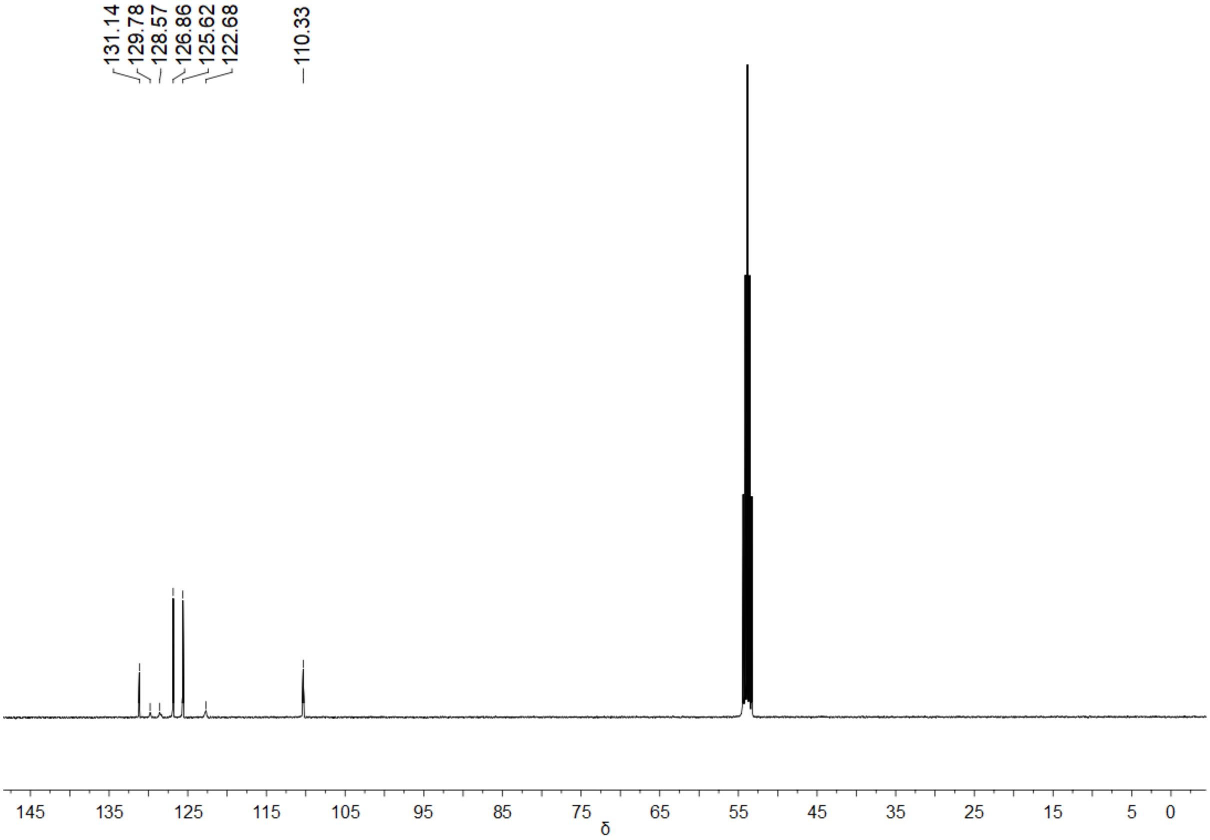

Figure S2. ^{13}C NMR spectrum of compound **3**

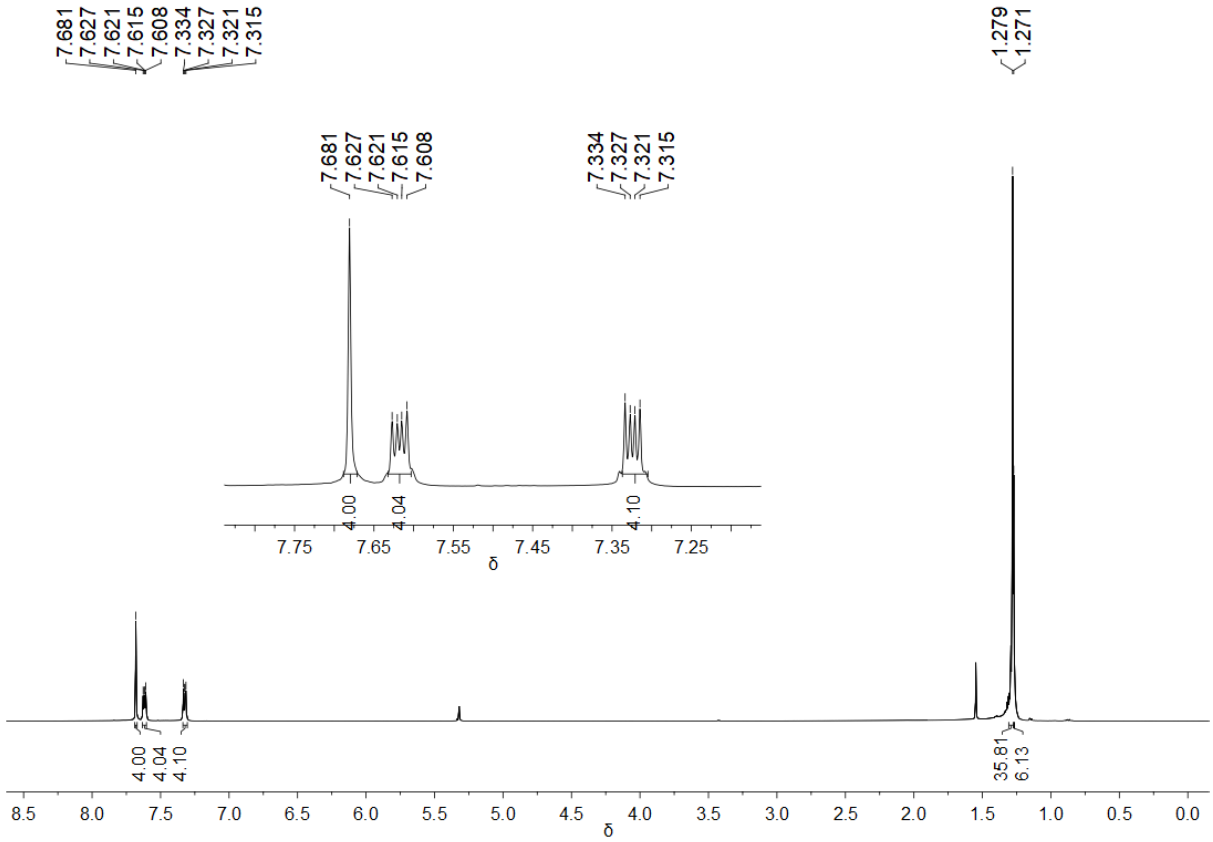

Figure S3. ^{1}H NMR spectrum of **2a**

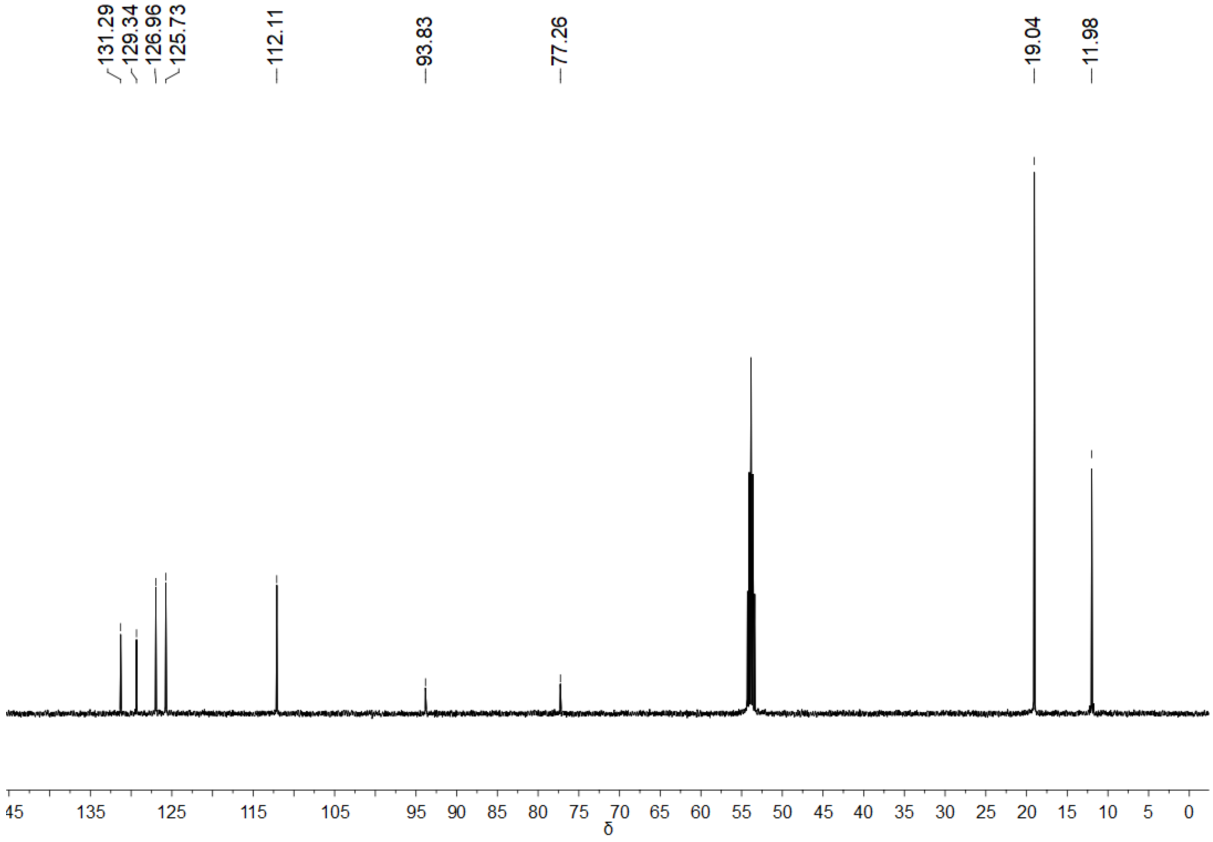

Figure S4. ^{13}C NMR spectrum of **2a**

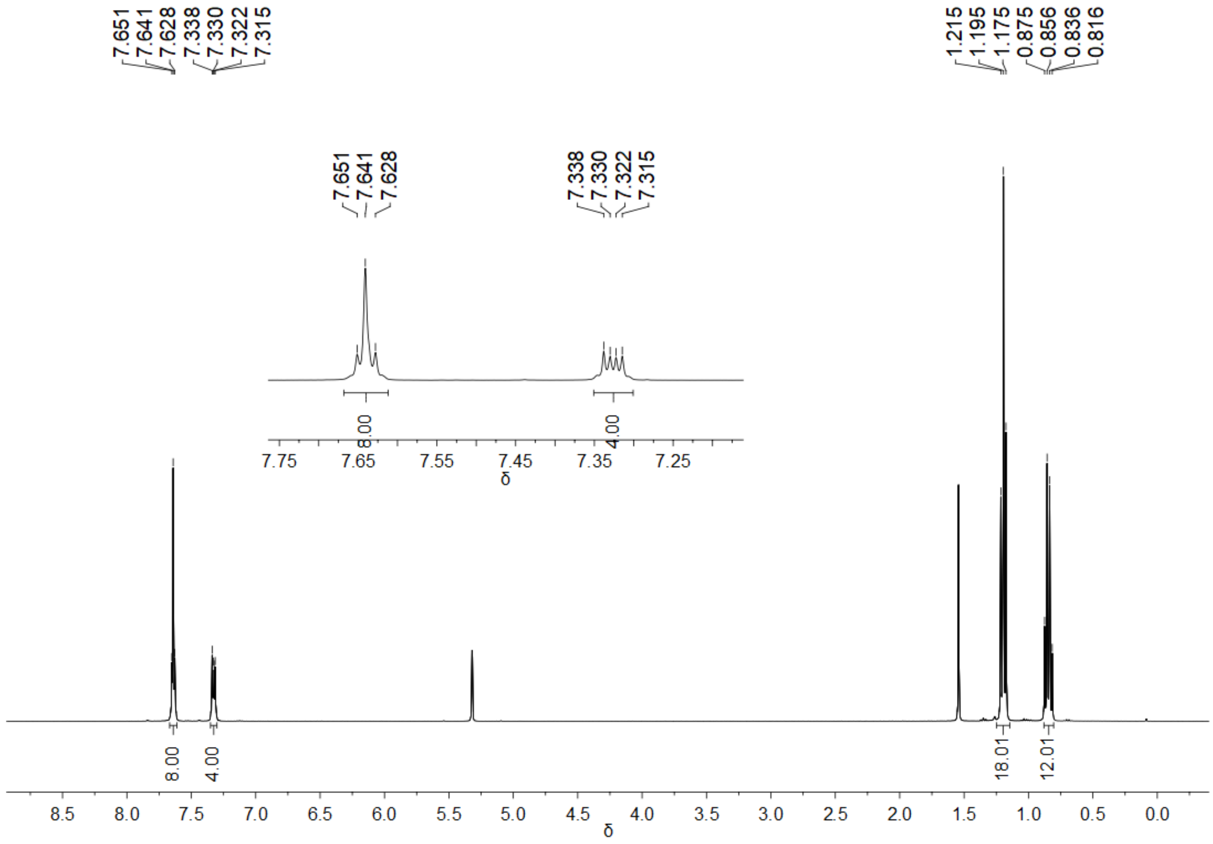

Figure S5. ^{1}H NMR spectrum of **2b**

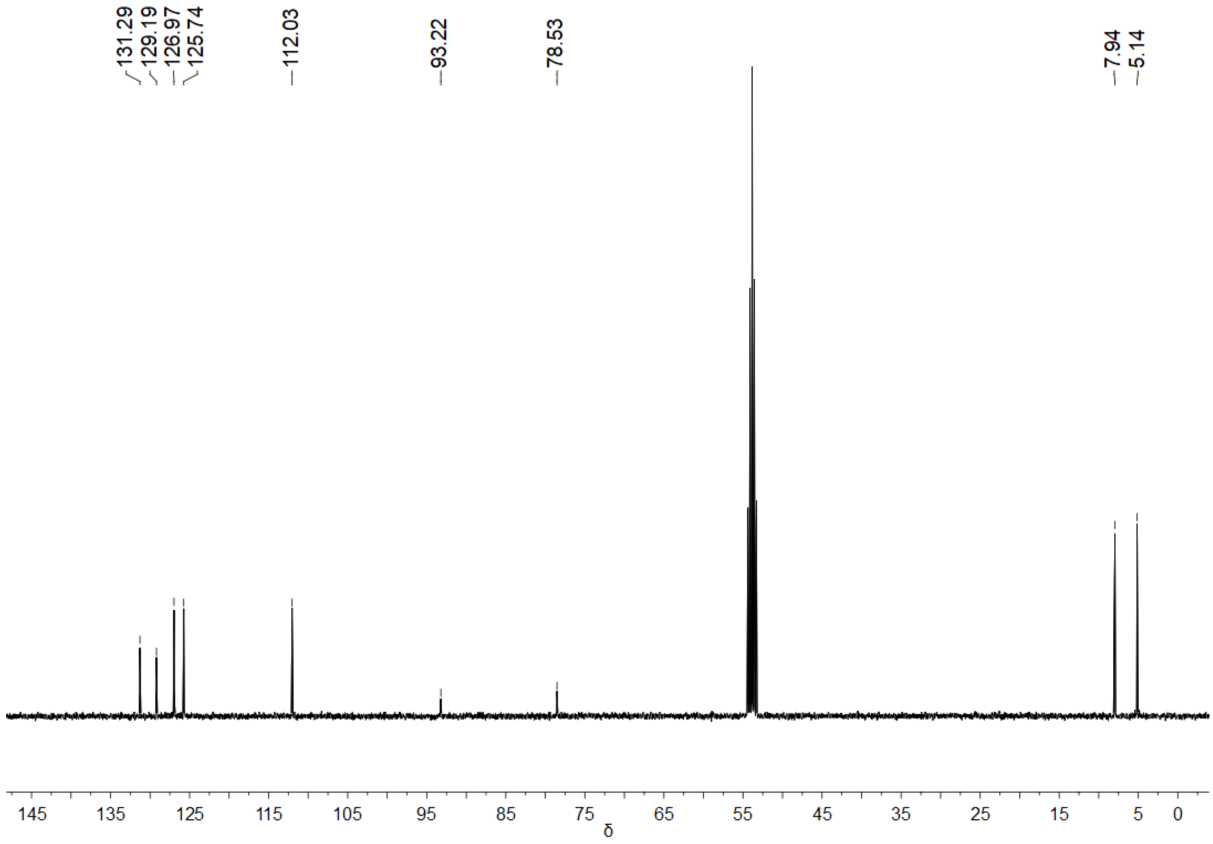

Figure S6. ^{13}C NMR spectrum of **2b**

2. Photophysical properties

UV-vis absorption spectra were recorded on a SHIMADZU UV-3600 plus UV-VIS-NIR spectrophotometer.

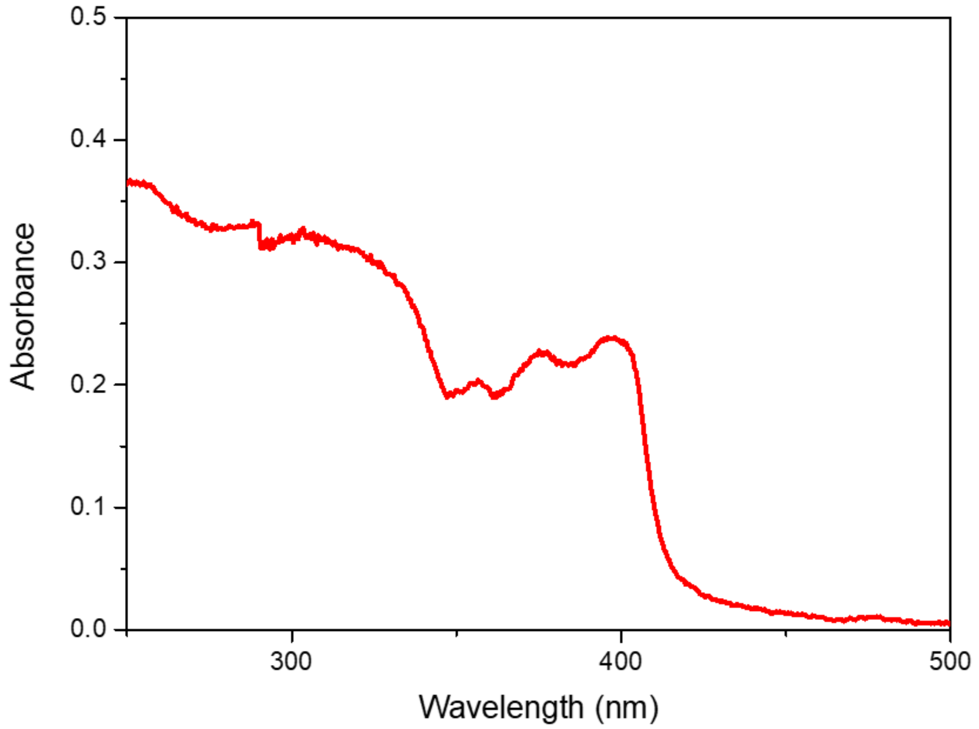

Figure S7. Absorption spectrum of a thin film of **2a** on quartz.

The absolute fluorescence quantum yield was measured using a FLS920P fluorescence spectrometer (Edinburgh Instrument) equipped with an integrating sphere with its inner face coated with BENFLEC®(Edinburgh Instrument). Spectral correction curves were provided by Edinburgh Instrument.

For the stability tests, solutions of **1a** and **2a** in toluene of identical concentration (1×10^{-5} mol/L) and volume were stored in 100 mL volumetric flasks under the same conditions. Prior to transfer into the flasks, the toluene solution was bubbled with air. The flasks were then sealed and placed on a bench under indoor lighting.

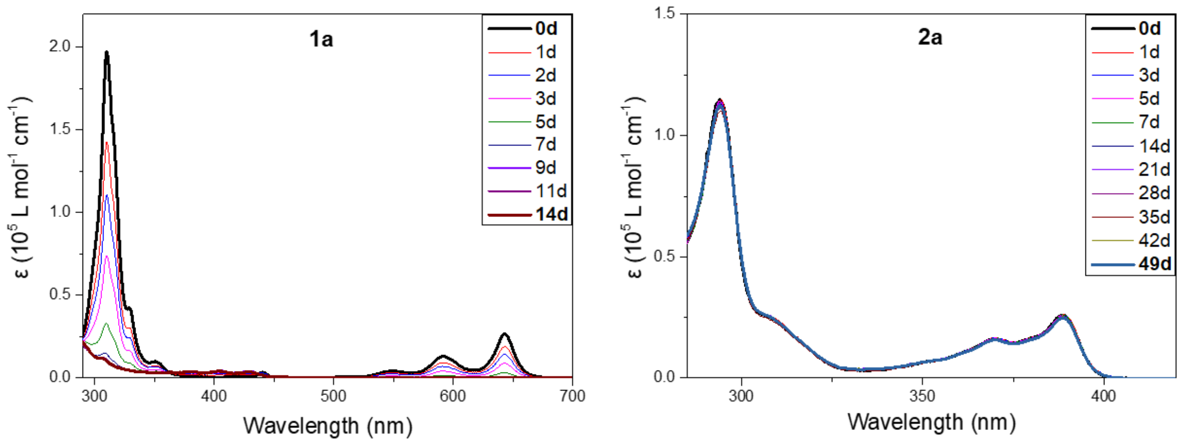

Figure S8. Change of UV/vis absorption spectra over time of **1a** (left) and **2a** (right) (1×10^{-5} mol/L in toluene).

3. Cyclic Voltammetry

Cyclic voltammetry was performed on a PAR Potentiostat / Galvanostat Model 263A Electrochemical Station (Princeton Applied Research) at a scan rate of 100 mV/s. Samples were dissolved in anhydrous CH_2Cl_2 with 0.1 M tetrabutylammonium hexafluorophosphate (Bu_4NPF_6) as supporting electrolyte. A platinum bead was used as a working electrode, a platinum wire was used as an auxiliary electrode, and a silver wire was used as a pseudo-reference. Ferrocene/ferrocenium was used as an internal standard.

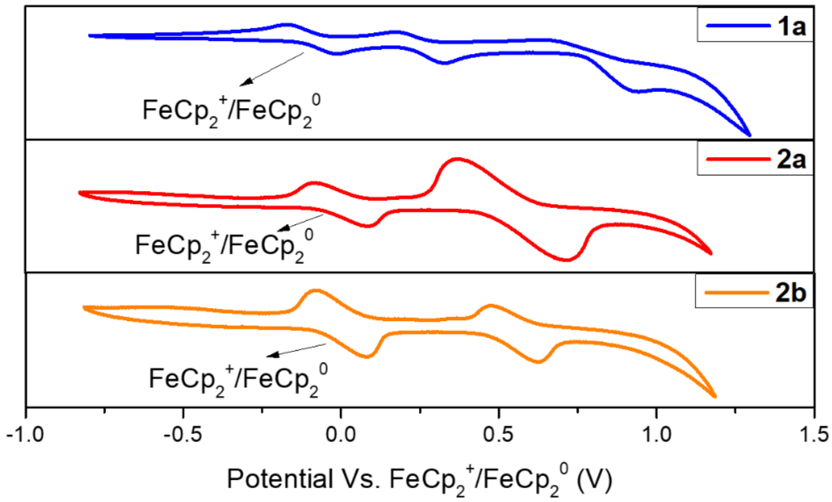

Figure S9. Cyclic voltammograms of **1a**, **2a** and **2b** recorded in CH_2Cl_2 with $FeCp_2^+/FeCp_2^0$ as the internal standard.

Table S1. Oxidation potentials, experimental and calculated frontier molecular orbital energy levels of **1a** and **2a**.

	Experimental				Calculated	
Molecule	E_{ox1}[a] (V)	HOMO[b] (eV)	λ_{edge} (nm)	ΔE_{opt}[c] (eV)	HOMO[d] (eV)	LUMO[d] (eV)
**1a**	0.37	−5.47	672	1.84	−4.91	−2.95
**2a**	0.54	−5.64	402	3.08	−5.20	−1.39

[a] Half-wave potential versus ferrocene/ferrocenium for the first oxidation wave; [b] Estimated from HOMO = −5.10 − E_{ox1} (eV); [c] ΔE_{opt} was estimated from λ_{edge}; [d] Calculated at the B3LYP level of DFT with 6-311++G(d,p) basis set.

4. X-ray crystallography

X-ray crystallography data were collected on a Bruker D8 Venture Diffractometer.

Table S2. Summary of crystallographic data for four compounds.

	**1a**	**2a**	**1b**	**2b**
Space Group	$P-1$	$P-1$	$P-1$	$P-1$
Unit Cell Length (Å)	a=7.5650(15)<br>b=7.7500(15)<br>c=16.835(3)	a=7.4461(4)<br>b=7.7756(4)<br>c=16.8204(9)	a=7.249(5)<br>b=10.263(8)<br>c=11.421(9)	a=7.235(2)<br>b=10.135(3)<br>c=11.368(5)
Unit Cell Angels (deg)	α=89.15(3)<br>β=78.42(3)<br>γ=83.63(3)	α=88.582(2)<br>β=78.370(2)<br>γ=84.786(2)	α=81.44(2)<br>β=89.54(2)<br>γ=81.83(2)	α=82.285(12)<br>β=89.283(11)<br>γ=80.990(10)
Cell Volume (Å^3)	960.927	949.905	831.615	815.881
R-factor (%)	4.86	6.15	5.52	6.35
Packing Motif	2D brickwork	2D brickwork	1D columnar	1D columnar
π-π distance (Å)	3.403; 3.319	3.362; 3.358	3.458	3.422

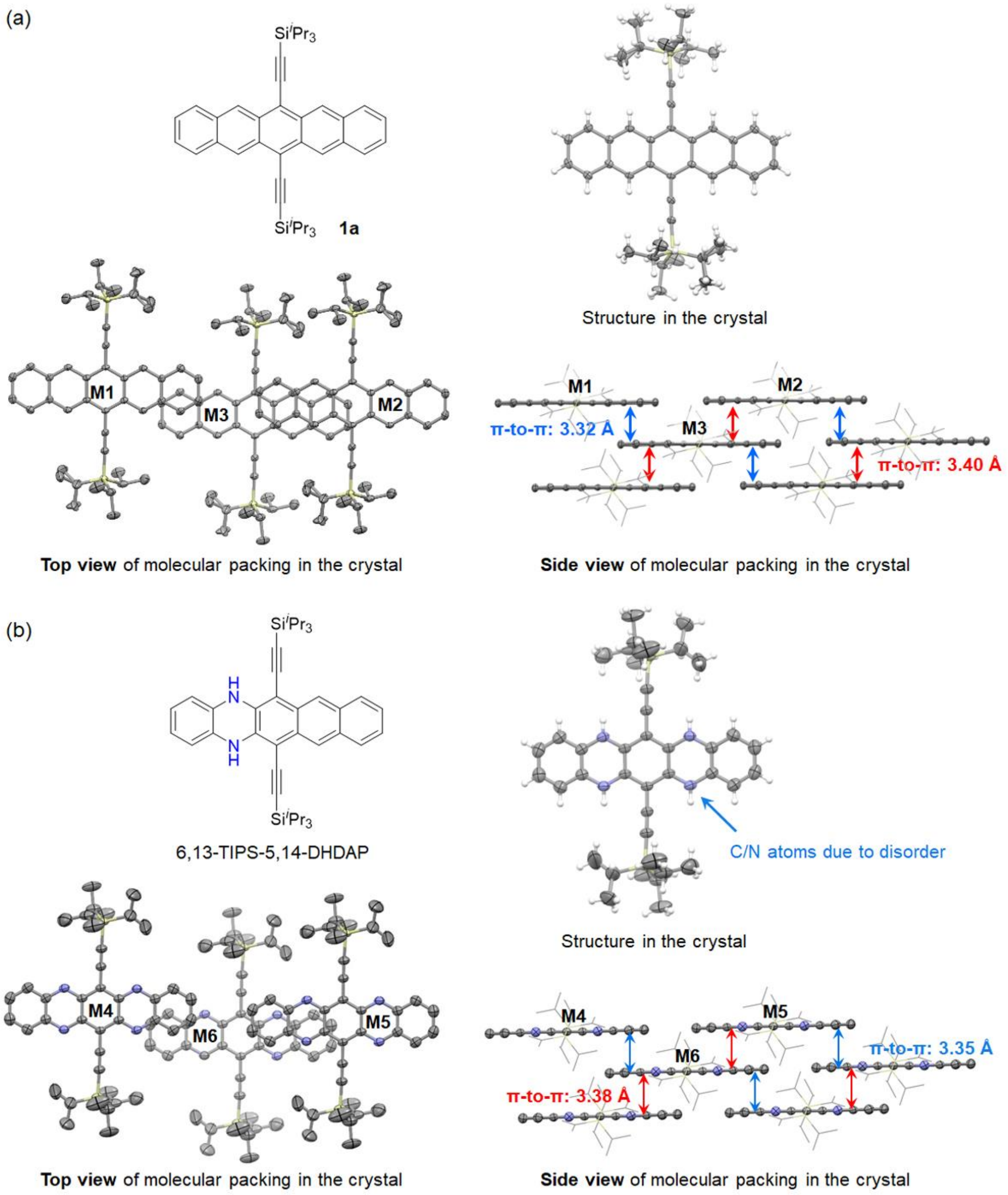

Figure S10. (a) The structure and molecular packing of 6,13-bis(triisopropylsilylethynyl)-pentacene (**1a**) in the crystal; (b) the structure and molecular packing of 6,13-bis(triisopropylsilylethynyl)-5,14-dihydro-5,14-diazapentacene (6,13-TIPS-5,14-DHDAP) in the crystal. (Hydrogen atoms are removed for clarity, triisopropylsilylethynyl groups are shown as wires in the side view, and other atoms are shown as ellipsoids set at 50% probability.)

5. Computational studies

The electron structure is calculated at DFT/b3lyp [2] level with the 6-311++g(d, p) [3,4,5,6,7,8,9,10,11] basis set using Gaussian 16 Revision B.01[12]. The reorganization energy is calculated with an optimized structure at the same level as single point calculation regardless of the thermal correction. For other properties (HOMO, LUMO, charge transfer integral), the geometries under consideration were extracted from the crystal structures obtained from the experiment.

The intramolecular reorganization energy is calculated using Nelson's 4-point methods as equation **B.1**.[13]

$$\lambda_e = \lambda_1 + \lambda_2 = \left[E_{\text{Neutral}}^{opt} - E_{\text{Neutral}}^{\text{Vertical}}\right] + \left[E_{\text{Charged}}^{\text{opt}} - E_{\text{Charged}}^{\text{Vertical}}\right] \quad \textbf{B.1}$$

The calculation of charge transfer integral V follows the methods introduced by Valeev in 2006,[14] as equation **B.2**.

$$V_{eff} = \frac{J_{ij} - 0.5(e_i + e_j)S_{ij}}{1 - S_{ij}^2} \quad \textbf{B.2}$$

$$e_{i(j)} = \langle \Phi_{i(j)} | H | \Phi_{i(j)} \rangle$$

$$S_{ij} = \langle \Phi_i \mid \Phi_j \rangle$$

$$J_{ij} = \langle \Phi_i | H | \Phi_j \rangle$$

Here, Φ_i/Φ_j is the HOMO/LUMO of the monomer.

The Marcus hopping rate is calculated according to equation **B.3**:

$$k_{i\to f} = \frac{2\pi V^2}{h}\left(\frac{\pi}{\lambda k_B T}\right)^{0.5} \exp\left(-\frac{(w_f + \lambda)^2}{4k_B T\lambda}\right) \quad \textbf{B.3}$$

Here, w_f is the Gibbs free energy of the carrier transfer process.

The carrier mobility is calculated using equation **B.4** derived from Einstein's relation.[15]

$$\mu_\phi = \frac{e}{2k_B T}\sum_i K_i r_i^2 P_i \cos^2\gamma_i \cos^2(\theta_i - \phi) \quad \textbf{B.4}$$

Here, K_i is the transfer integral associated with the specific pathway, r_i is the distance between dimer along the transfer pathway, and P_i is the corresponding hopping possibility.

Table S3. Hole reorganization energies of **1a**, **2a**, **1b** and **2b** calculated using Nelson four points method at B3LYP/6-311++G(d,p).

Molecule	Reorganization energy (meV)		
	λ_1^+	λ_2^+	λ^+
**1a**	68.38	68.13	136.51
**2a**	81.87	76.24	158.11
**1b**	69.54	69.35	138.89
**2b**	83.39	80.77	164.16

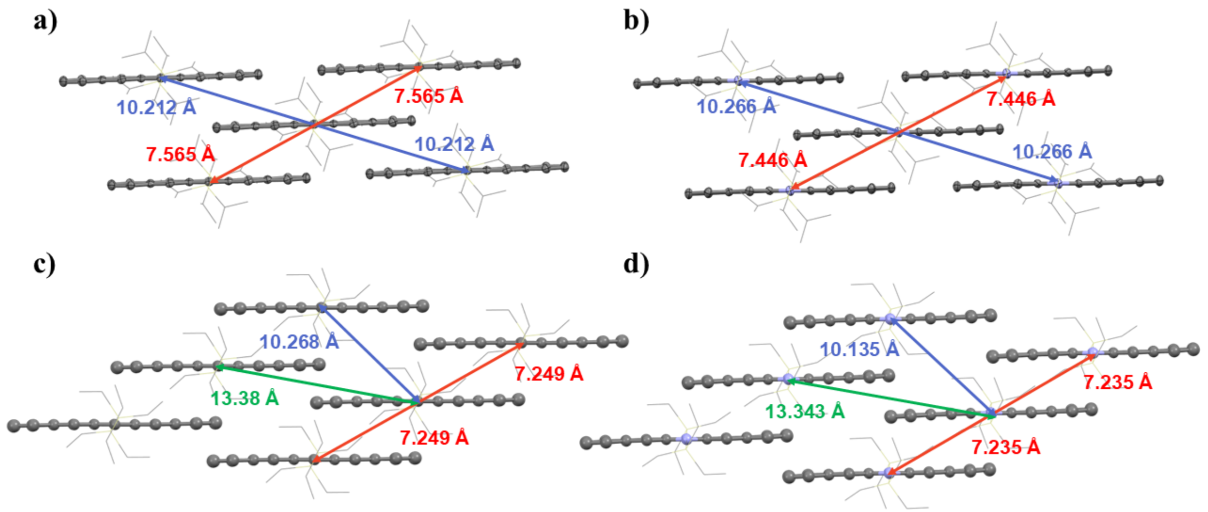

Figure S11. The hole transport pathways along with the π-stacked directions in the crystal structures of **1a** (a), **2a** (b), **1b** (c) and **2b** (d) for calculating transfer integrals. (Hydrogen atoms are removed for clarity, triisopropylsilylethynyl groups are shown as wires, and other atoms are shown as ellipsoids set at 50% probability. The numbers mark the distances between the centroids of two neighboring molecules.)

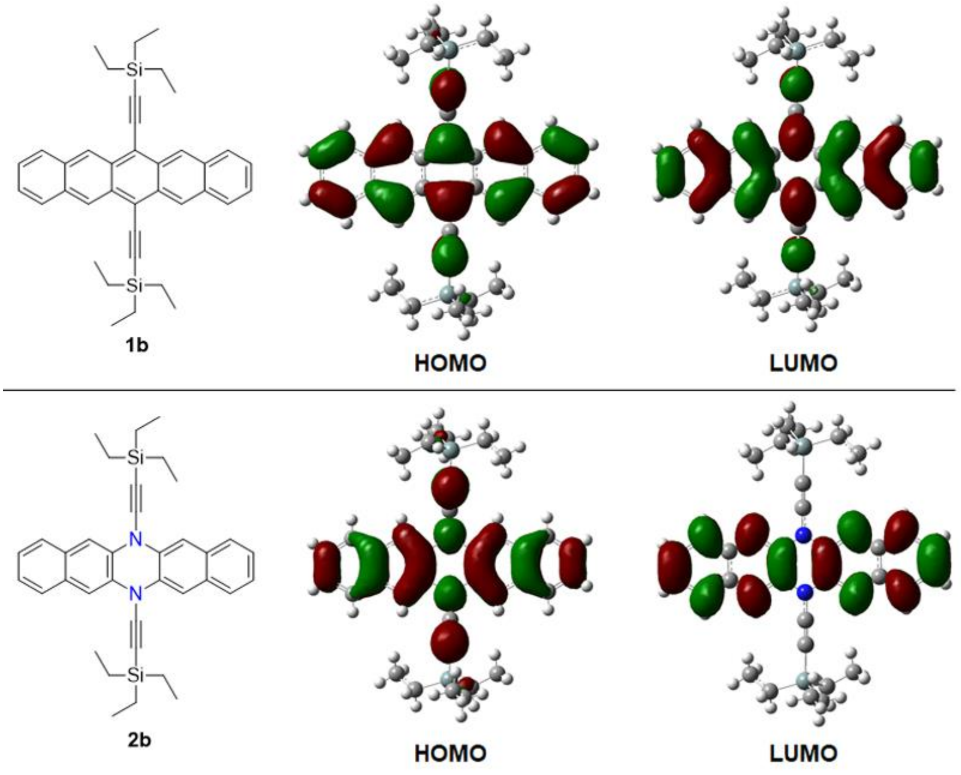

Figure S12 The frontier molecular orbitals of **1b** and **2b** calculated at the B3LYP level of DFT with 6-311++G(d, p) basis set.

6. Fabrication of organic field effect transistors (OFETs)

(1) Fabrication of thin films by dip coating

Dip-coated films were prepared on silicon substrates layered with 100 nm-thick thermally grown silica (SiO_2), solution-processed alumina (AlO_x), and a self-assembled monolayer (SAM) of either 12-cyclohexyldodecylphosphonic acid (CDPA) or 12-methoxydodecylphosphonic acid (MODPA) as composite dielectric materials. The $AlO_x/SiO_2/Si$ substrates were prepared following the reported procedures, [16] and then modified with CDPA and MODPA following the reported procedures. [16, 17] Dip-coating was performed by immersing the substrate into a solution of the designated compound and withdrawing it at a constant speed using a LongerPump TJ-3A syringe pump. The optimized conditions for each material are summarized in the table below.

Compound	Solvent (v : v)	Concentration (mg/mL)	pulling speed (µm/min)	SAM used for modification of alumina
**1a**	CH_2Cl_2 : acetone (1 : 1)	1.0	47	CDPA
**1b**	CH_2Cl_2 : methanol (3 : 1)	0.8	47	MODPA
**2a**	CH_2Cl_2 : methanol (2 : 1)	1.5	47	CDPA
**2b**	CH_2Cl_2 : methanol (3 : 1)	1.2	55	MODPA

The dip-coated films were placed in a vacuum oven overnight to completely remove solvent residues. To form top-contact source and drain electrodes, a 40 nm-thick layer of gold was deposited through a shadow mask onto the organic films using an Edward Auto 306 vacuum deposition system at a pressure of 4.0×10^{-6} torr.

(2) Fabrication of thin films by bar coating

Bar-coated films of **1a** and **2a** were prepared on highly doped silicon substrates coated with 300 nm SiO_2 and divinyltetramethyldisiloxane bis(benzocyclobutene) (BCB) successively. The temperature of the substrate was set at 35 °C and stabilized for 5 min before coating. The distance between the bar and the BCB-coated substrate was 100 µm. 8 µL of a solution of **1a** or **2a** in 1,3-dichloropropane (4 mg/mL) was injected into the gap. After 5 seconds, the coating equipment was activated to move the substrate with a constant speed 8 µm/s.

For the preparation of films using **1b** and **2b**, the temperature of the substrate was set at 55 °C and stabilized for 5 min before coating. The distance between the bar and the BCB-coated substrate was maintained at 100 µm. 8 µL of a solution of **1b** or **2b** in anisole (4 mg/mL) was injected into the gap. After 5 seconds, the coating equipment was activated to move the substrate with a constant speed 12 µm/s.

OFETs were constructed in a top contact, bottom gate configuration by transferring 80-nm-thick gold stripes onto the bar-coated films to form source and drain electrodes. The BCB-coated 300-nm-thick SiO_2/Si served as the gate insulator and gate electrode of the OFETs.

7. Characterization of thin films and OFETs

(1) Reflected polarized-light microscopy

Polarized optical images of organic thin films were obtained using a Nikon 50iPOL Microscope or Nikon Eclipse LV100 POL.

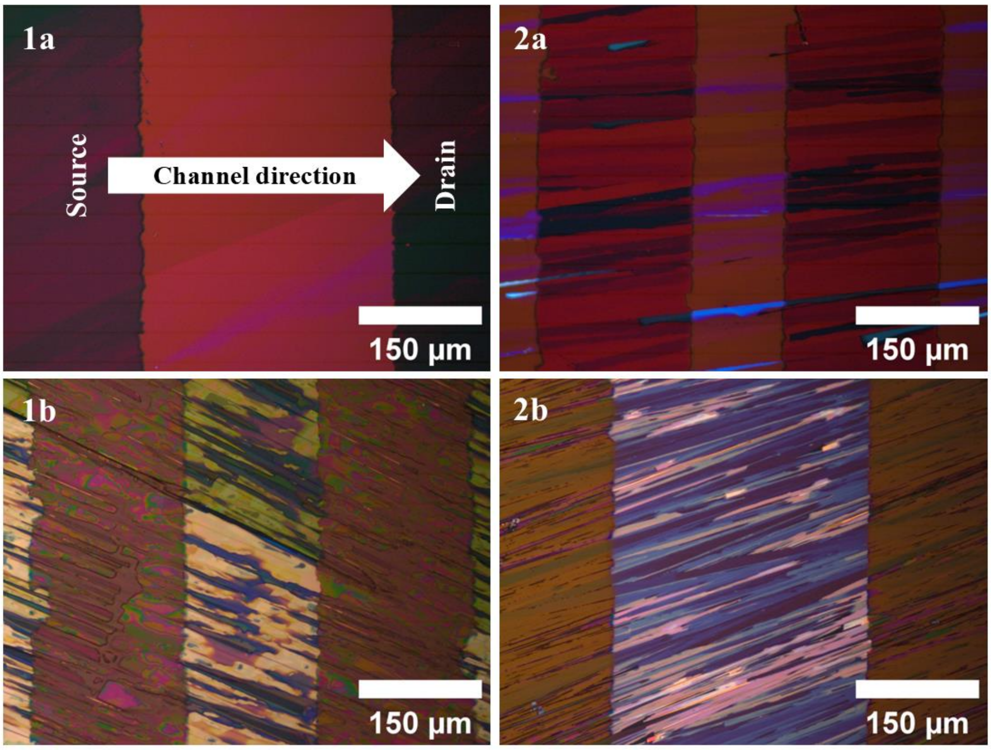

Figure S13. Reflected polarized-light micrographs for thin films of **1a** and **2a** on CDPA-modified AlOx/SiO_2/Si substrates and those of **1b** and **2b** on MODPA-modified AlOx/SiO_2/Si substrate with top-contact gold electrodes.

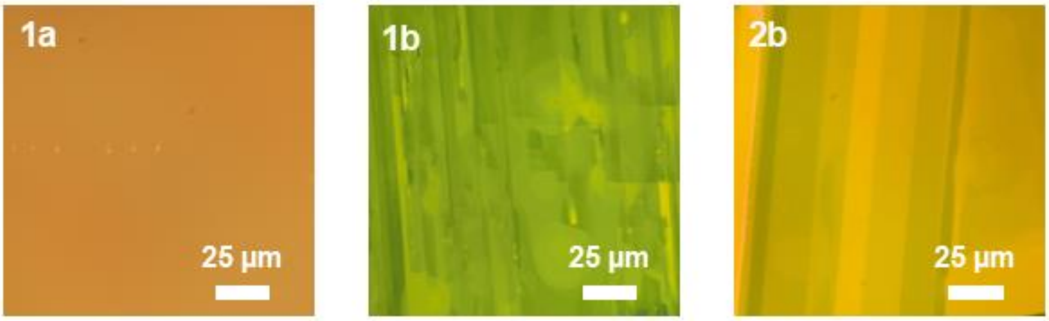

Figure S14. Reflected polarized-light micrographs for bar-coated thin films of **1a**, **1b** and **2b**.

(2) Atomic force microscopy (AFM)

The AFM images were collected with a Nanoscope IIIa Multimode Microscope (Digital Instruments) or Veeco Nanoscope 3D using tapping mode and in air under ambient conditions.

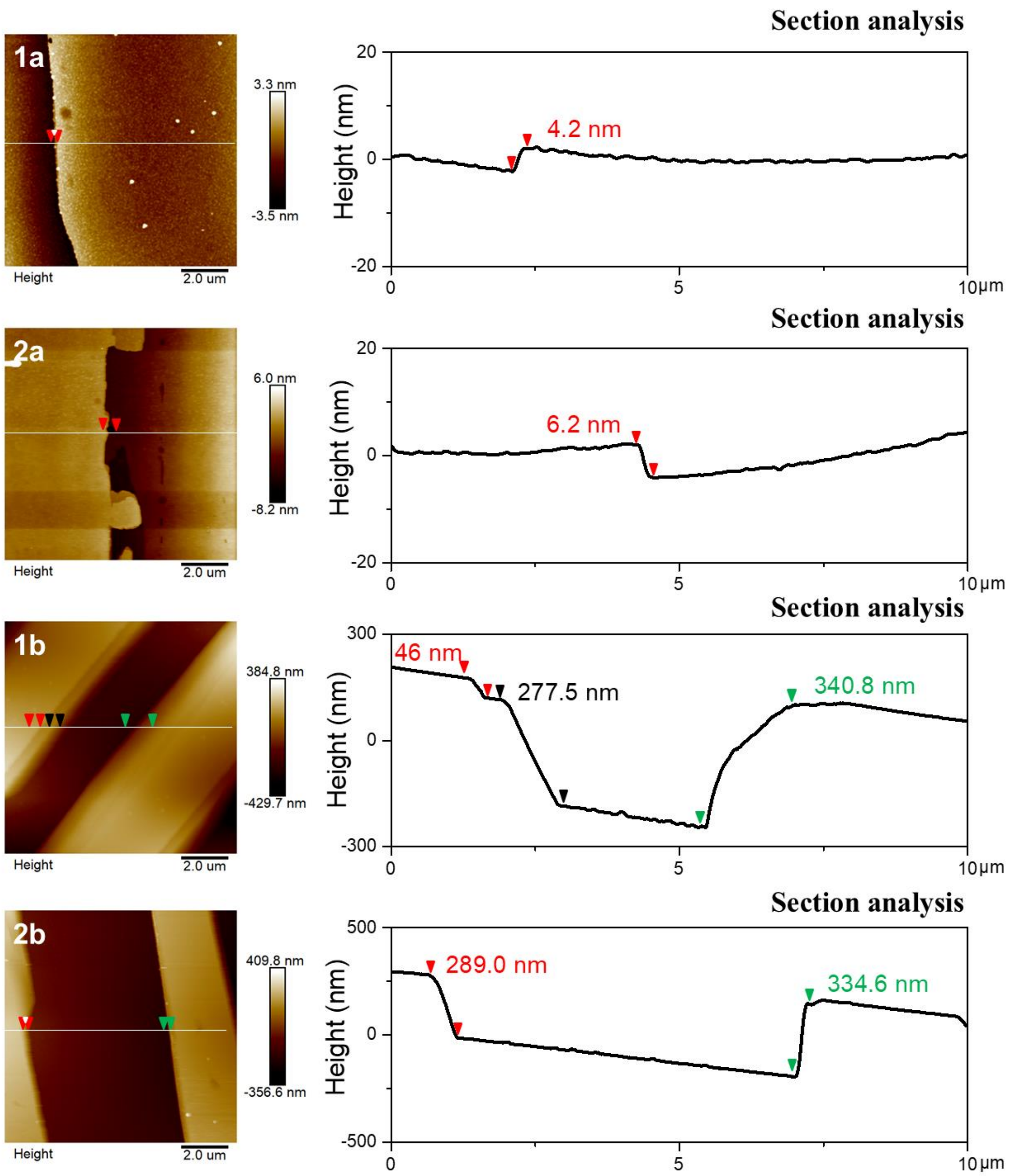

Figure S15. AFM section analysis for dip-coated films of **1a**, **2a** on CDPA-modified AlOx/SiO_2/Si substrate and dip-coated films of **1b**, **2b** on MODPA-modified AlOx/SiO_2/Si substrate.

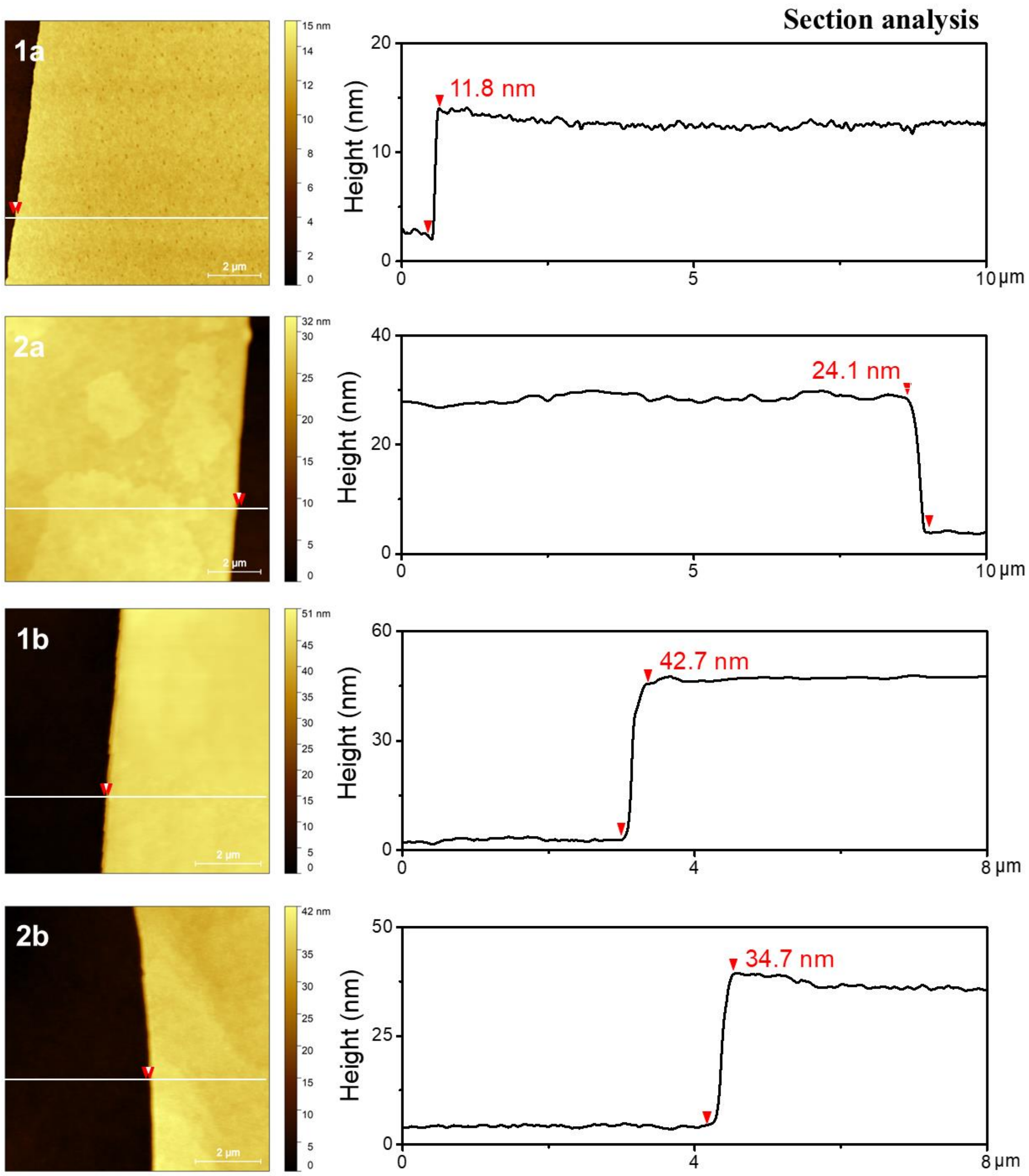

Figure S16. AFM section analysis for the bar-coated films of **1a**, **2a**, **1b** and **2b**.

(3) X-ray diffraction (XRD)

XRD data of organic thin films were recorded using a Rigaku SmartLab X-Ray Refractometer.

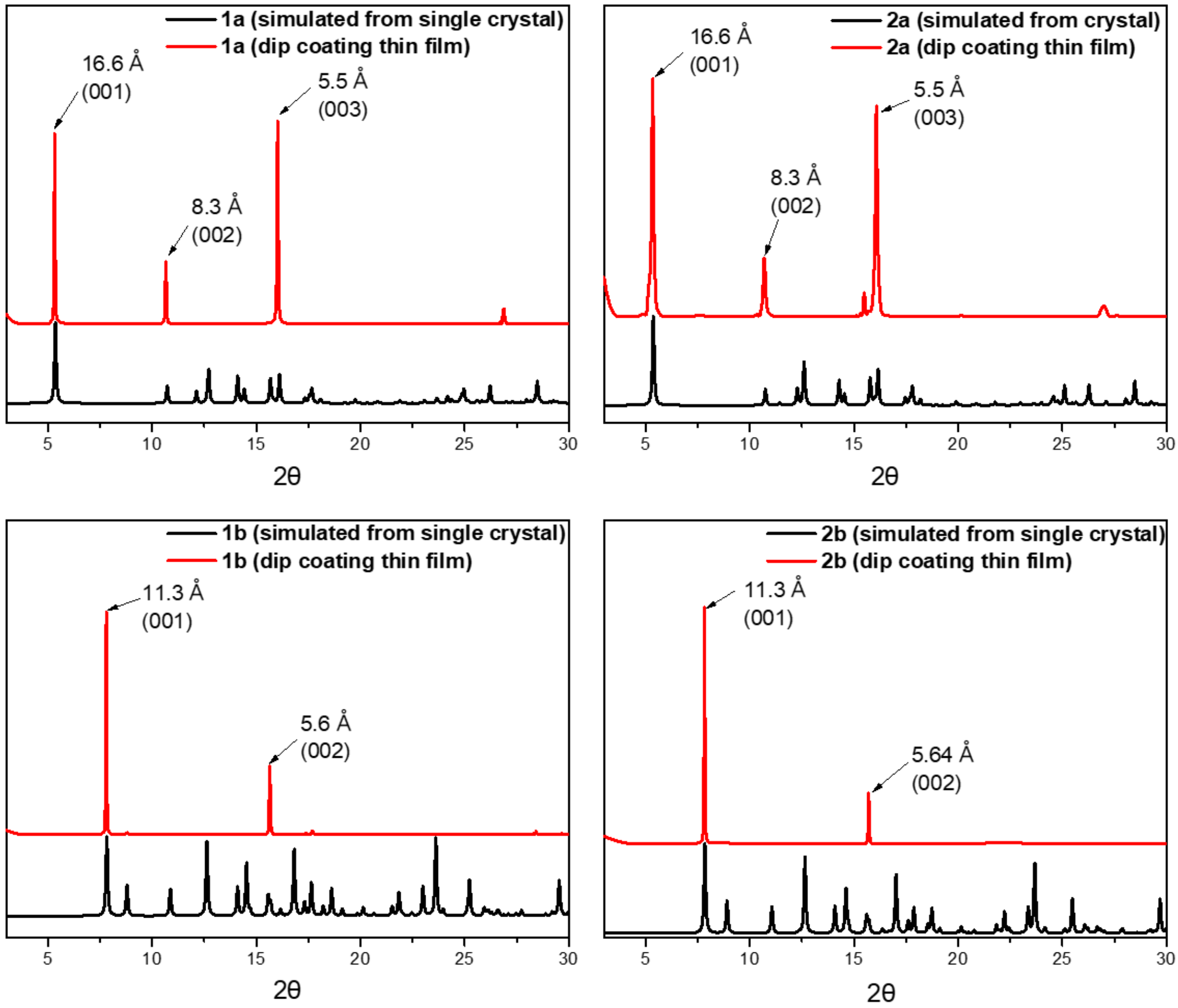

Figure S17. X-ray diffractions of dip-coated thin films of **1a**, **2a** on CDPA-modified $AlO_x/SiO_2/Si$ substrate and **1b**, **2b** on MODPA-modified $AlO_x/SiO_2/Si$ substrate.

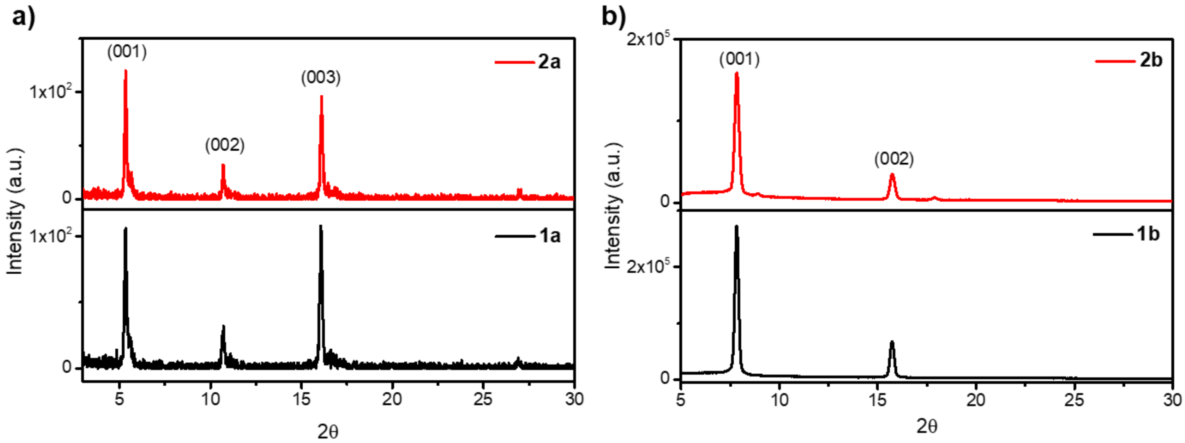

Figure S18. X-ray diffractions of bar-coated thin films of **1a** and **2a** (a), **1b** and **2b** (b).

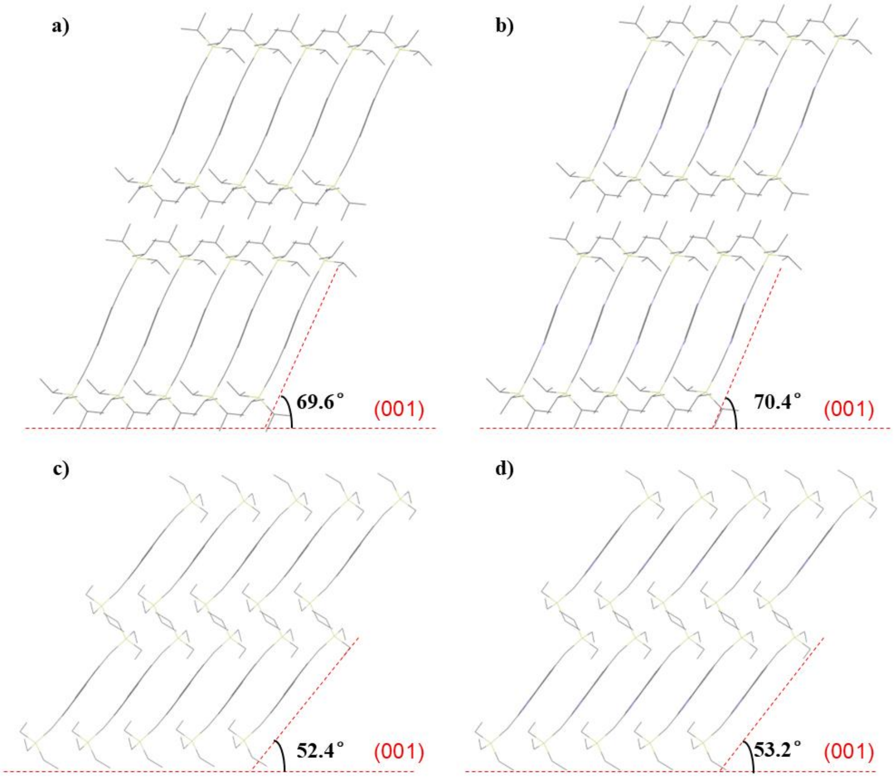

Figure S19. Orientation of **1a** (a), **2a** (b), **1b** (c), **2b** (d) with the (001) crystal plane parallel to the substrate surface.

(4) Grazing incidence wide-angle X-ray Scattering (GIWAXS)

GIWAXS data were carried out with a Xeuss 2.0 SAXS/WAXS laboratory beamline using a Cu X-ray source (8.05 keV, 1.54 Å) and Pilatus3R 300K detector. The incidence angle is 0.2 °.

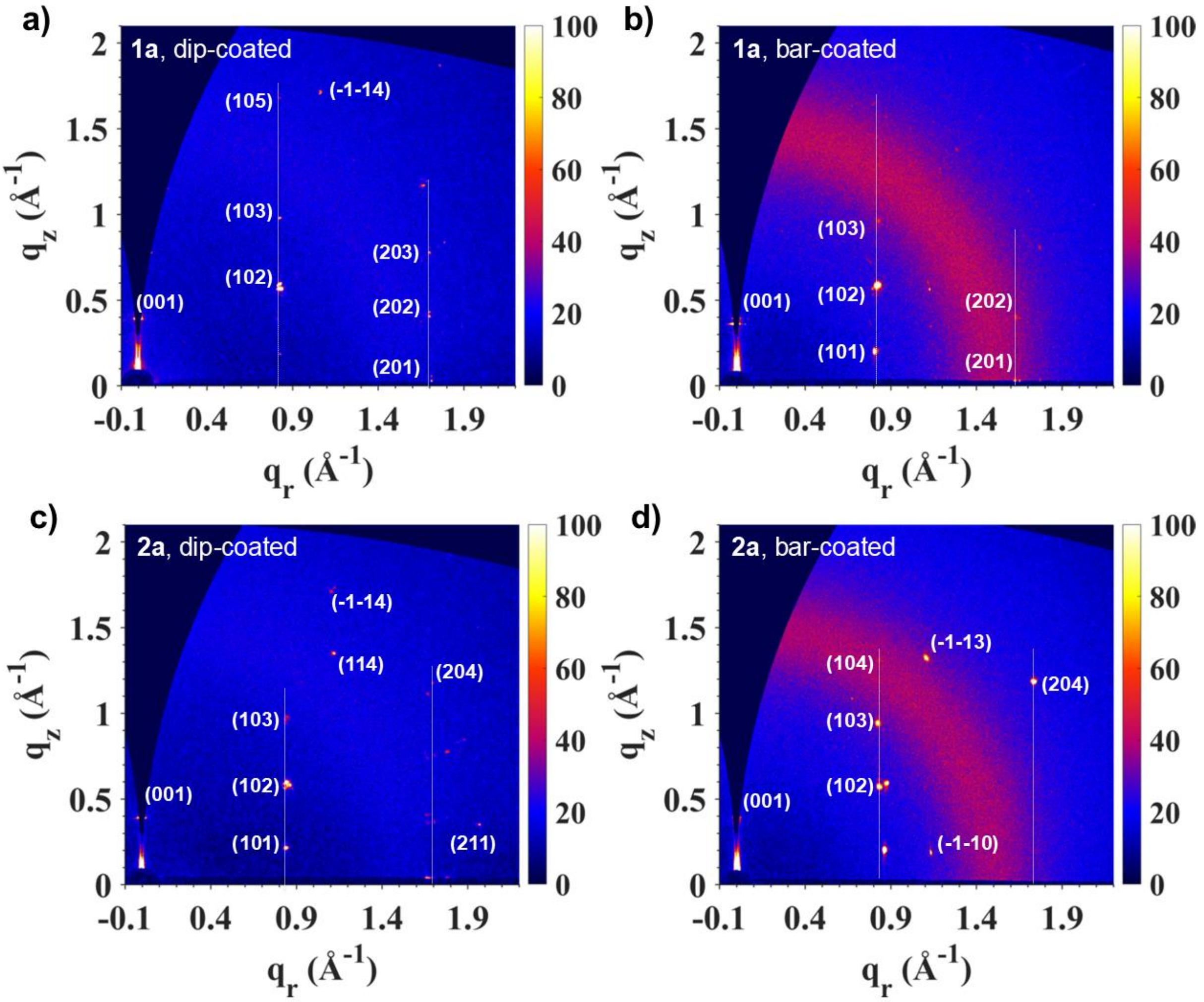

Figure S20. 2D GIWAXS patterns of thin films of **1a** and **2a** prepared by dip coated and bar-coated method. The incident X-ray beam direction was perpendicular to dip-coating (a and c) or bar-coating (b and d) directions.

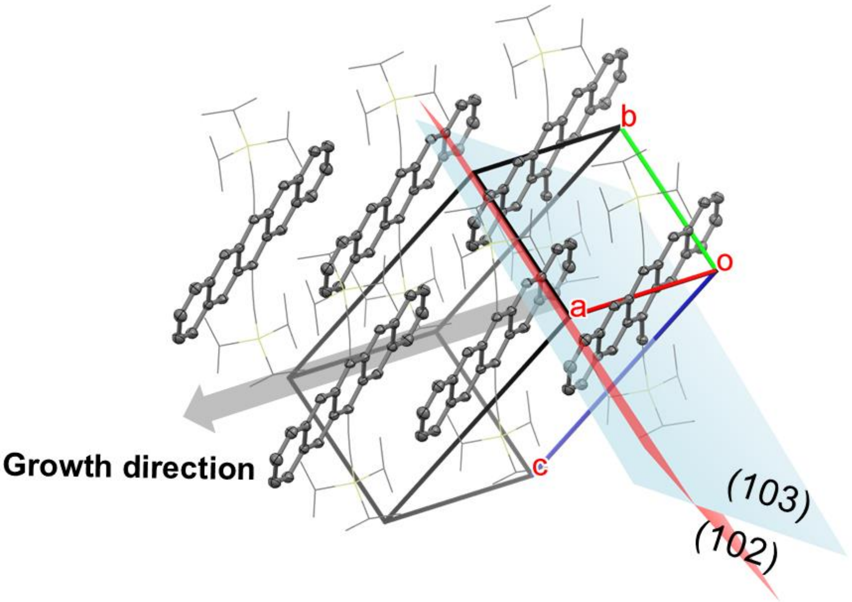

Figure S21. Growth direction of the crystal domains of **1a** derived from the GIWAXS patterns shown in Figure S20.

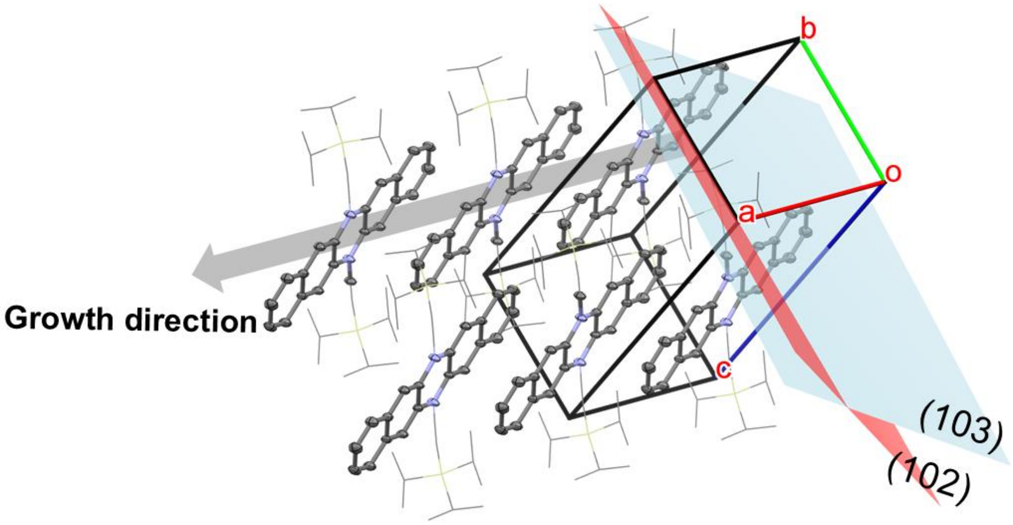

Figure S22. Growth direction of the crystal domains of **2a** derived from the GIWAXS patterns shown in Figure S20.

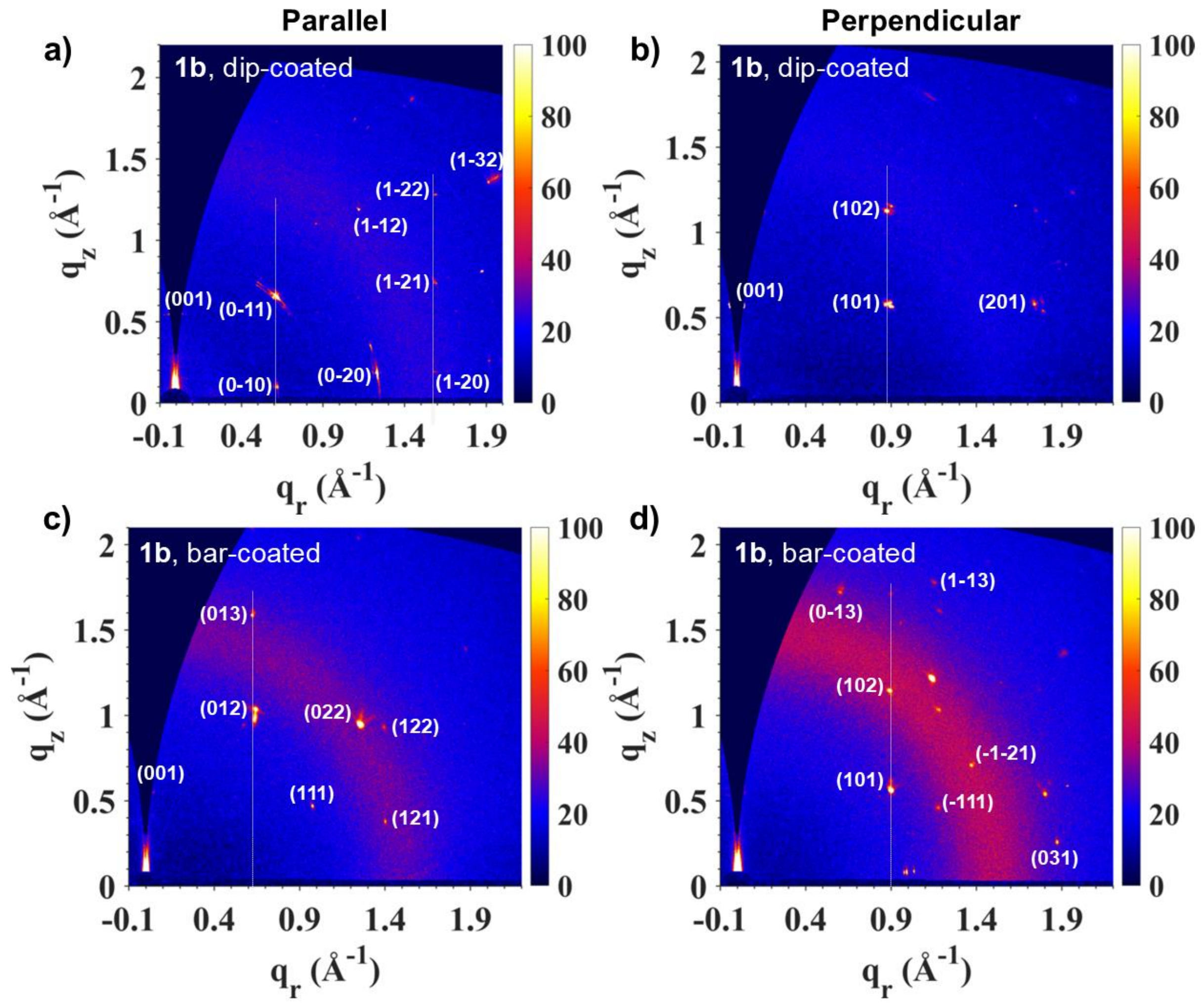

Figure S23. 2D GIWAXS patterns of dip coated or bar-coated thin films of **1b**. The incident X-ray beam directions were parallel (a and c) and perpendicular (b and d) with dip-coated (a and b) or bar-coated (c and d) direction. (The wide arcs in Panels c and d are due to the SiO_2 under the bar-coated thin films.)

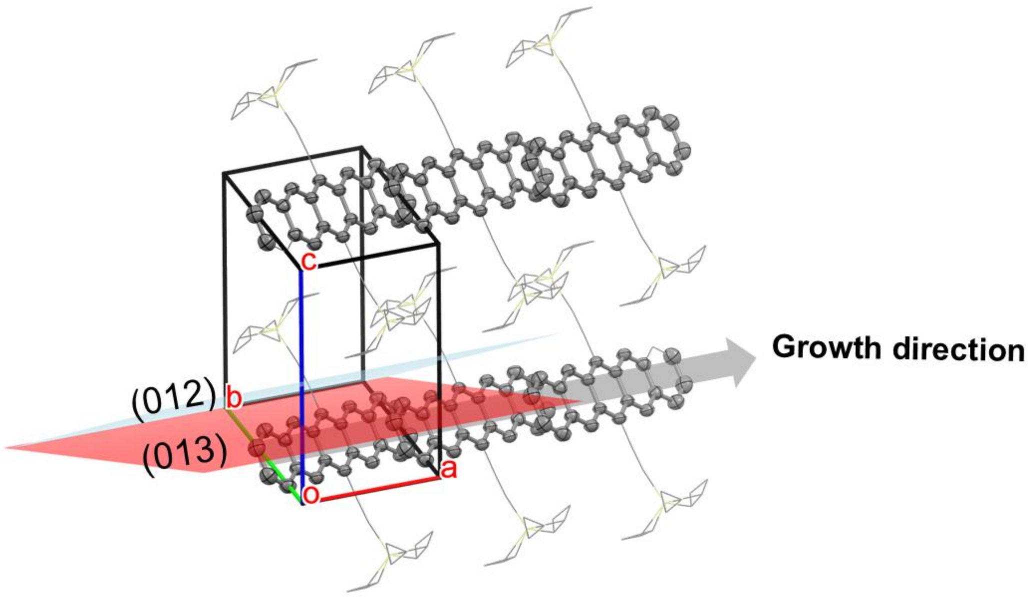

Figure S24. Growth direction of the crystal domains of **1b** in the bar-coated films derived from the GIWAXS patterns shown in Figure S23c.

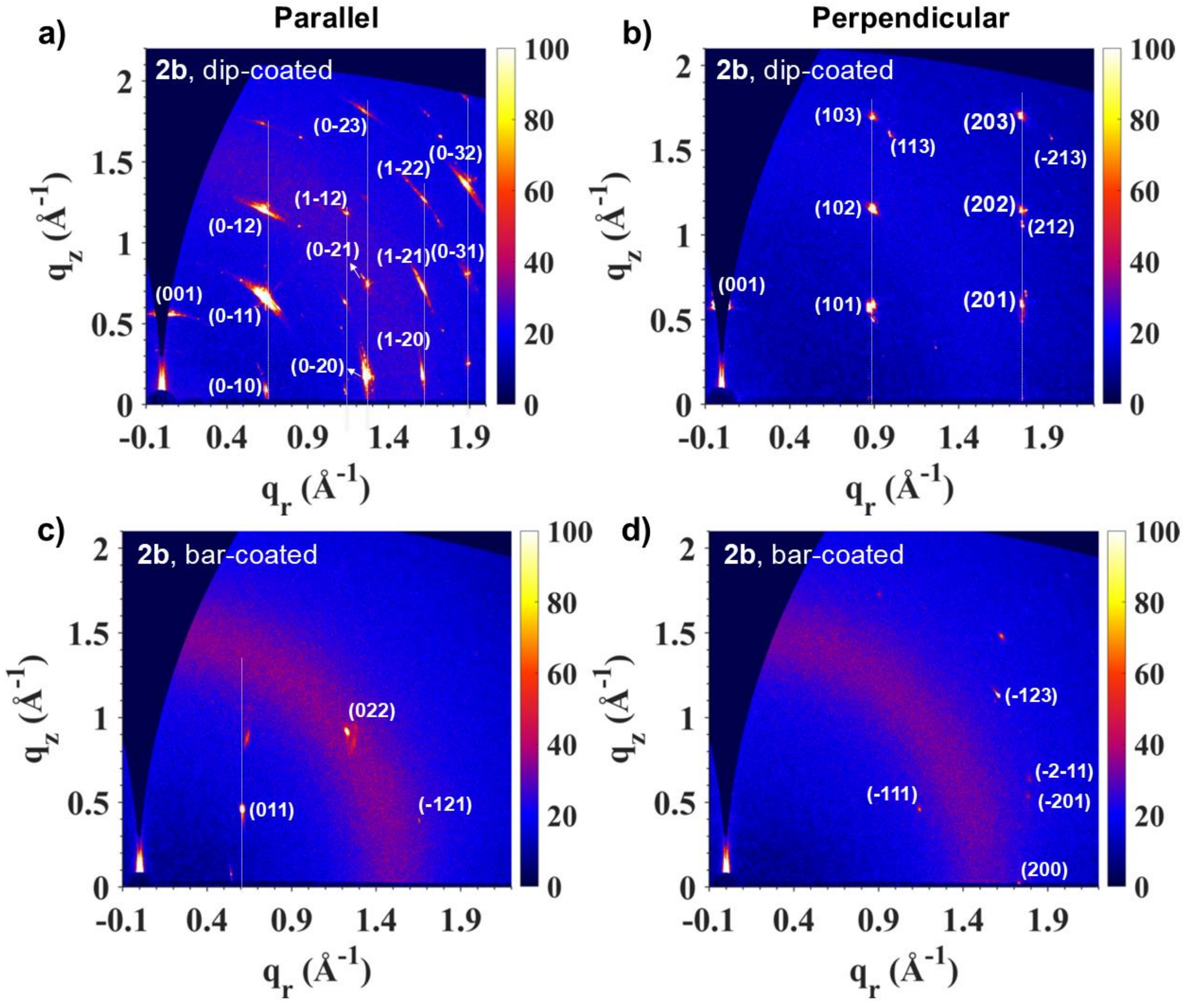

Figure S25. 2D GIWAXS patterns of dip coated and bar-coated thin films of **2b**. The incident X-ray beam directions were parallel (a and c) and perpendicular (b and d) with dip-coated (a and b) or bar-coated (c and d) direction. (The wide arcs in Panels c and d are due to the SiO_2 under the bar-coated thin films.)

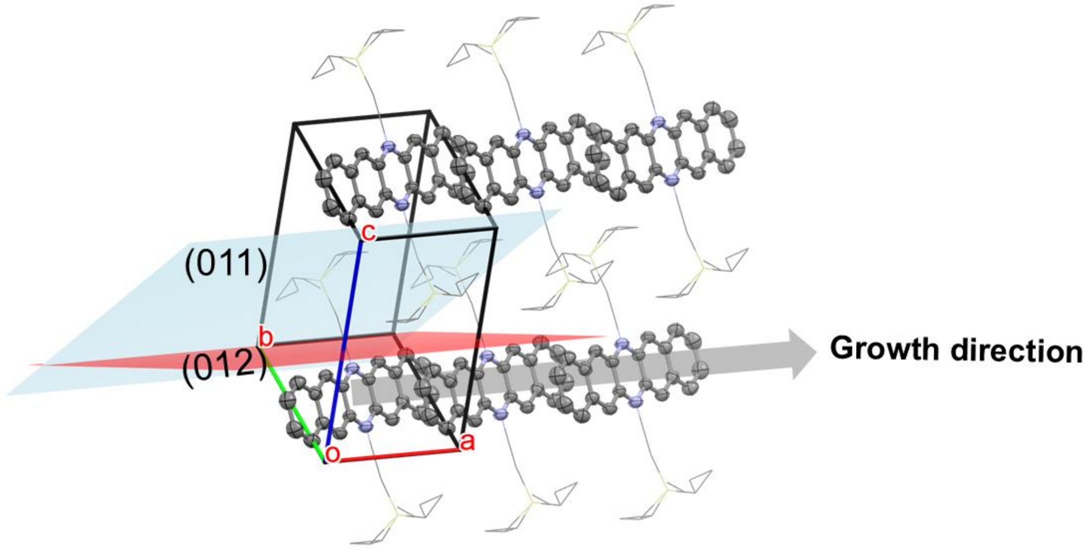

Figure S26. Growth direction of the crystal domains of **2b** in the bar-coated films derived from the GIWAXS patterns shown in Figure S25c.

(5) Electrical measurements

The dip-coated OFETs were characterized using a JANIS ST-500 Micromanipulated Probe Station and a Keithley 4200 Semiconductor Characterization System in ambient air at room temperature. The exact channel width (W) and length (L) for mobility calculations were measured with a Nikon 50iPOL microscope, equipped with a SPOT Insight CCD camera (Diagnostic Instrument). The specific capacitance (C_i) of the CDPA or MODPA-modified AlOx/SiO_2 was 28 nF/cm^2.

The bar-coated OFETs were characterized in a nitrogen-filled glovebox using a Keysight B1500A semiconductor parameter analyzer. For mobility calculations, the W/L values were corrected using optical microscope images, and the specific capacitance of the BCB-SiO_2 was 11 nF/cm^2.

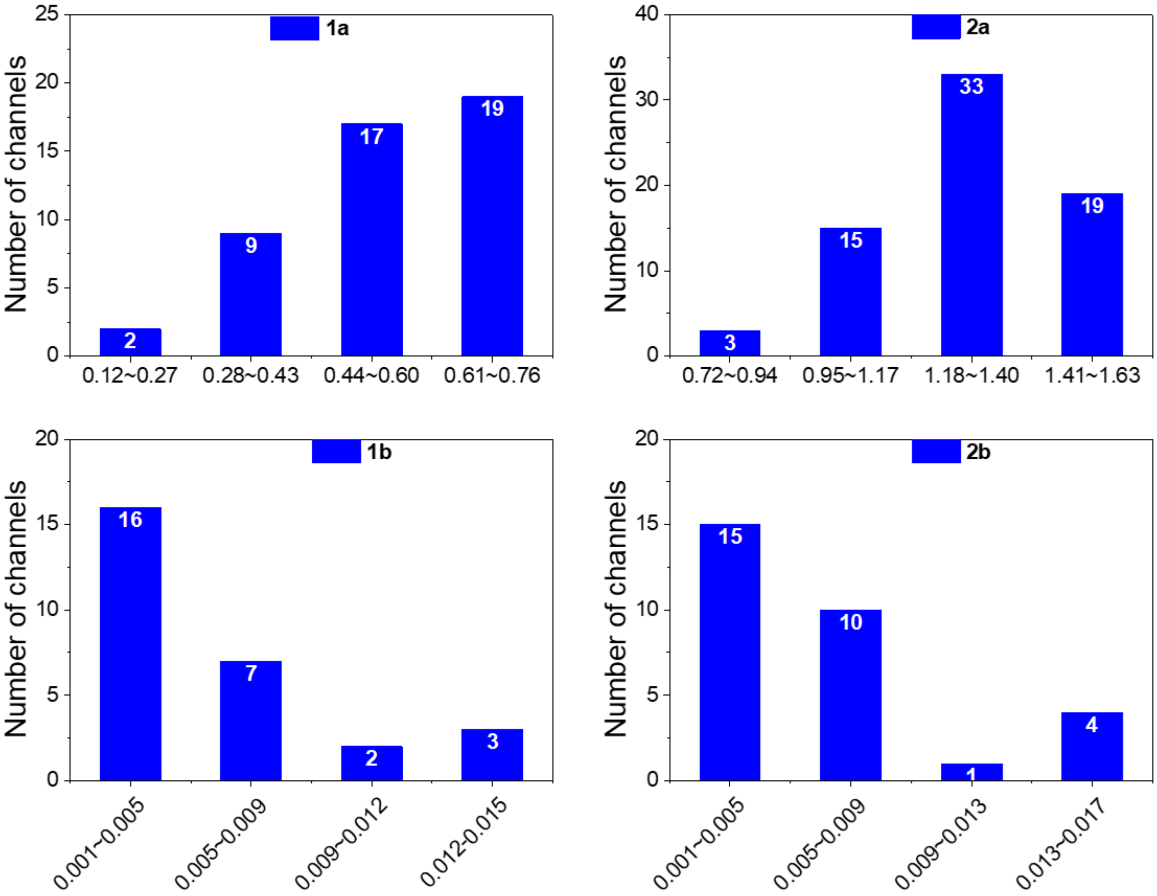

Figure S27. Statistic histograms for the field effect mobilities of dip-coated OFETs of **1a/b** and **2a/b**, measured from the saturation regime at room temperature.

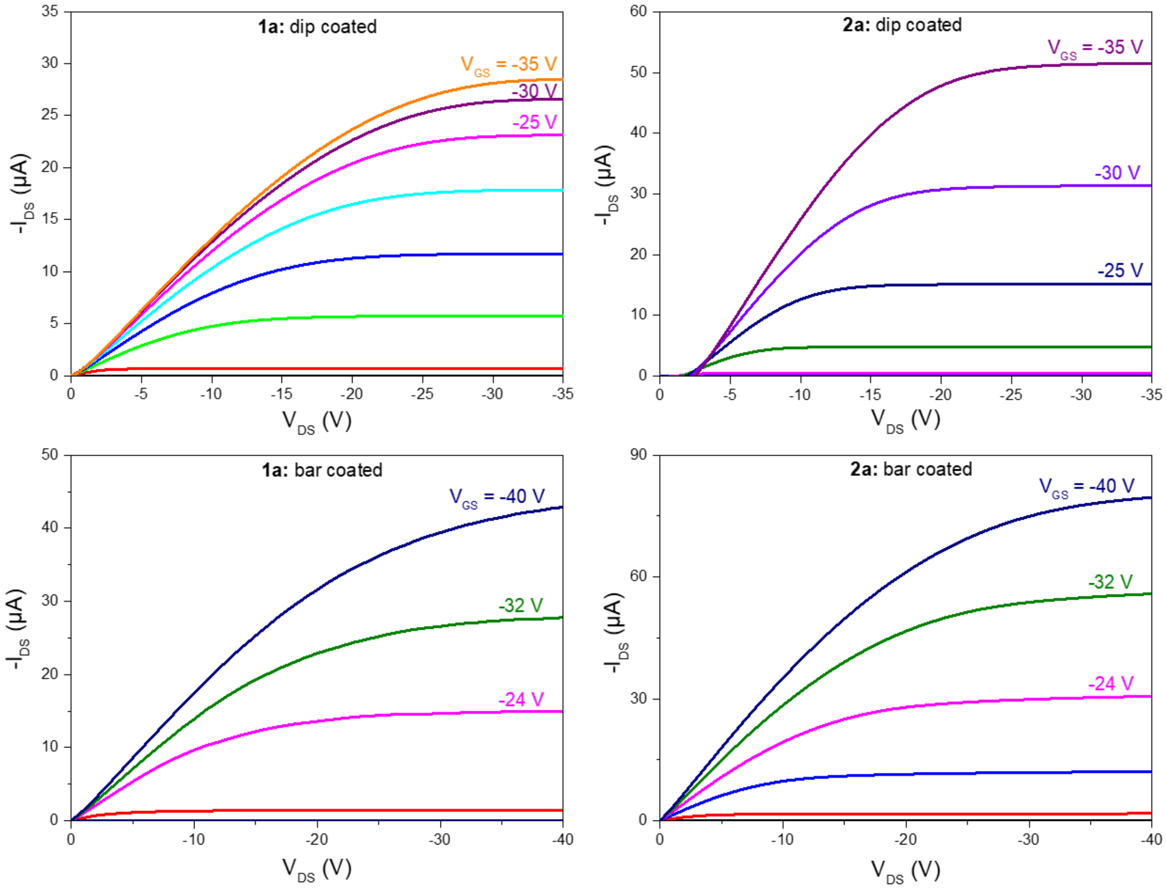

Figure S28. Output *I-V* curves of the dip-coated and bar-coated OFETs of **1a** and **2a**. (The dip-coated OFETs were measured in ambient air, and the bar-coated OFETs were measured under a N_2 atmosphere, both at room temperature.)

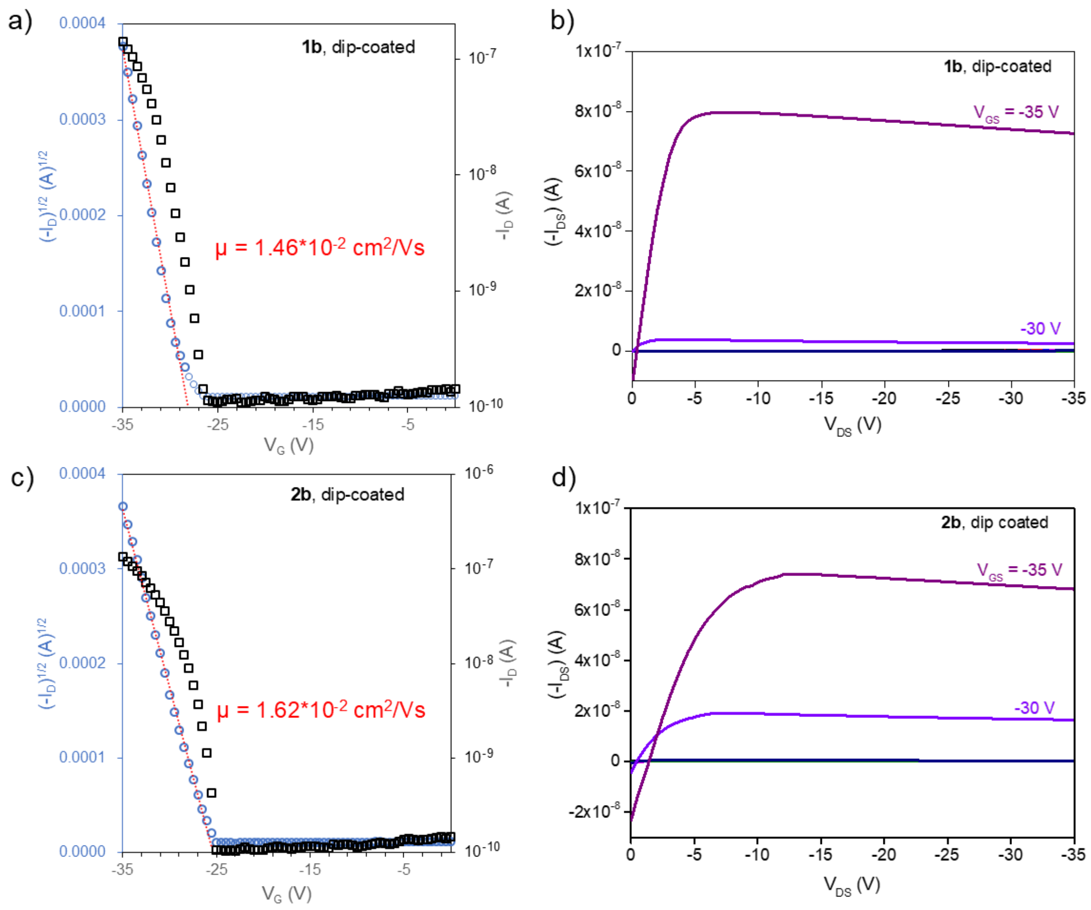

Figure S29. Transfer and output *I*–*V* curves measured from the best-performing dip-coated OFETs of **1b** (panels a and b, respectively) and **2b** (panels c and d, respectively), measured in ambient air at room temperature. (W/L is 14.5 for the OFET of **1b**, and 6.2 for **2b**, and C_i is 28 nF/cm^2)

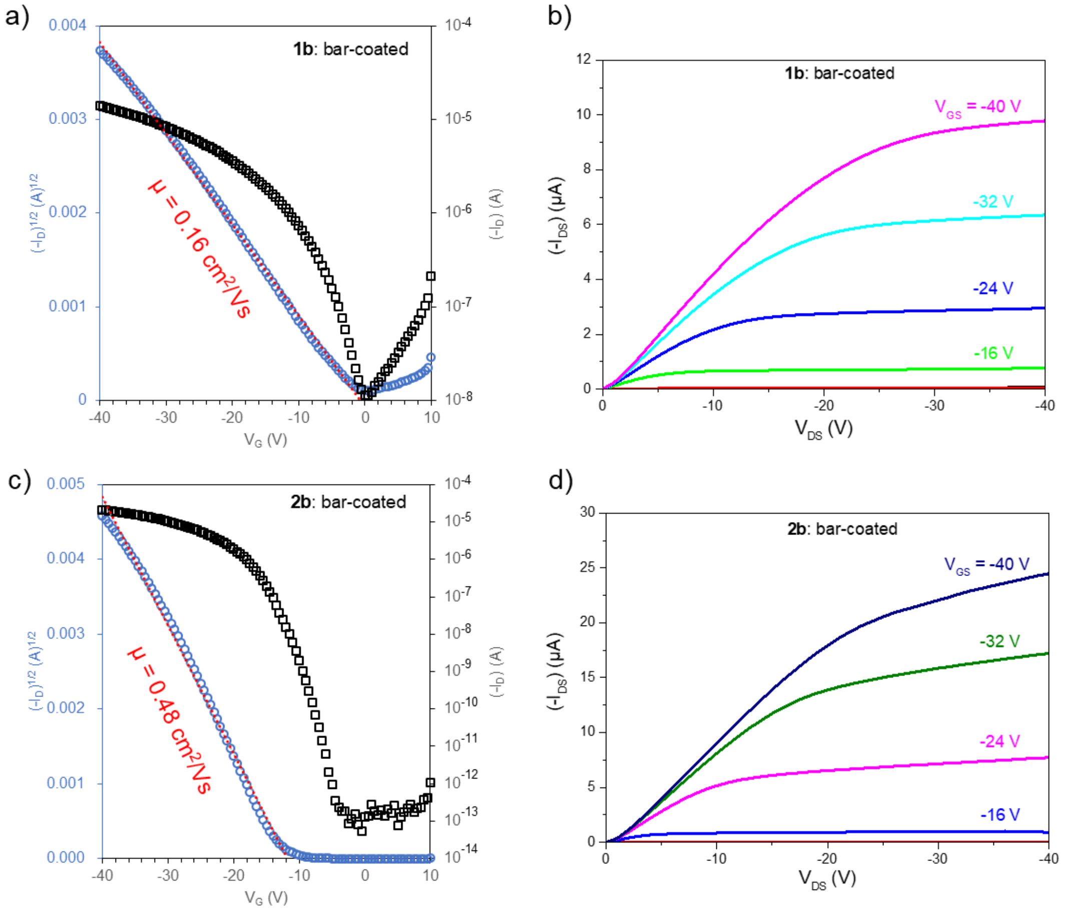

Figure S30. Transfer and output *I*–*V* curves measured from the typical bar-coated OFETs of **1b** (panels a and b, respectively) and **2b** (panels c and d, respectively), measured in N_2 atmosphere at room temperature. (W/L is 12.6 for the OFET of **1b**, and 14.4 for **2b**, and C_i is 11 nF/cm^2).

8. References

[1] Miao, Q.; Nguyen, T.-Q.; Someya, T.; Blanchet, G. B.; Nuckolls, C., Synthesis, Assembly, and Thin Film Transistors of Dihydrodiazapentacene: An Isostructural Motif for Pentacene. *J. Am. Chem. Soc.* **2003,** *125*, 10284–10287.

[2] Becke, A. D., A new mixing of Hartree–Fock and local density-functional theories. *J. Chem. Phys.* **1993,** *98*, 1372–1377.

[3] Spitznagel, G. W.; Clark, T.; von Ragué Schleyer, P.; Hehre, W. J., An evaluation of the performance of diffuse function-augmented basis sets for second row elements, Na-Cl. *J. Comput. Chem.* **1987,** *8*, 1109–1116.

[4] McLean, A.; Chandler, G., Contracted Gaussian basis sets for molecular calculations. I. Second row atoms, Z= 11–18. *J. Chem. Phys.* **1980,** *72*, 5639–5648.

[5] Clark, T.; Chandrasekhar, J.; Spitznagel, G. W.; Schleyer, P. V. R., Efficient diffuse function-augmented basis sets for anion calculations. III. The 3-21+ G basis set for first-row elements, Li–F. *J. Comput. Chem.* **1983,** *4*, 294–301.

[6] Ditchfield, R.; Hehre, W. J.; Pople, J. A., Self-consistent molecular-orbital methods. IX. An extended Gaussian-type basis for molecular-orbital studies of organic molecules. *J. Chem. Phys.* **1971,** *54*, 724–728.

[7] Francl, M. M.; Pietro, W. J.; Hehre, W. J.; Binkley, J. S.; Gordon, M. S.; DeFrees, D. J.; Pople, J. A., Self-consistent molecular orbital methods. XXIII. A polarization-type basis set for second-row elements. *J. Chem. Phys.* **1982,** *77*, 3654–3665.

[8] Gordon, M. S.; Binkley, J. S.; Pople, J. A.; Pietro, W. J.; Hehre, W. J., Self-consistent molecular-orbital methods. 22. Small split-valence basis sets for second-row elements. *J. Am. Chem. Soc.* **1982,** *104*, 2797–2803.

[9] Hehre, W. J.; Ditchfield, R.; Pople, J. A., Self—consistent molecular orbital methods. XII. Further extensions of Gaussian—type basis sets for use in molecular orbital studies of organic molecules. *J. Chem. Phys.* **1972,** *56*, 2257–2261.

[10] Krishnan, R.; Binkley, J. S.; Seeger, R.; Pople, J. A., Self-consistent molecular orbital methods. XX. A basis set for correlated wave functions. *J. Chem. Phys.* **1980,** *72*, 650–654.

[11] Hariharan, P. C.; Pople, J. A., The influence of polarization functions on molecular orbital hydrogenation energies. *Theor. Chim. Acta.* **1973,** *28*, 213–222.

[12] Frisch, M. J.; Trucks, G. W.; Schlegel, H. B.; Scuseria, G. E.; Robb, M. A.; Cheeseman, J. R.; Scalmani, G.; Barone, V.; Petersson, G. A.; Nakatsuji, H.; Li, X.; Caricato, M.; Marenich, A. V.; Bloino, J.; Janesko, B. G.; Gomperts, R.; Mennucci, B.; Hratchian, H. P.; Ortiz, J. V.; Izmaylov, A. F.; Sonnenberg, J. L.; Williams; Ding, F.; Lipparini, F.; Egidi, F.; Goings, J.; Peng, B.; Petrone, A.; Henderson, T.; Ranasinghe, D.; Zakrzewski, V. G.; Gao, J.; Rega, N.; Zheng, G.; Liang, W.; Hada, M.; Ehara, M.; Toyota, K.; Fukuda, R.; Hasegawa, J.; Ishida, M.; Nakajima, T.; Honda, Y.; Kitao, O.; Nakai, H.; Vreven, T.; Throssell, K.; Montgomery Jr., J. A.; Peralta, J. E.; Ogliaro, F.; Bearpark, M. J.; Heyd, J. J.; Brothers, E. N.; Kudin, K. N.; Staroverov, V. N.; Keith, T. A.; Kobayashi, R.; Normand, J.; Raghavachari, K.; Rendell, A. P.; Burant, J. C.; Iyengar, S. S.; Tomasi, J.; Cossi, M.; Millam, J. M.; Klene, M.; Adamo, C.; Cammi, R.; Ochterski, J. W.; Martin, R. L.; Morokuma, K.; Farkas, O.; Foresman, J. B.; Fox, D. J. *Gaussian 16 Rev. B.01*, Wallingford, CT, **2016**.

[13] Nelsen, S. F.; Blackstock, S. C.; Kim, Y., Estimation of inner shell Marcus terms for amino nitrogen compounds by molecular orbital calculations. *J. Am. Chem. Soc.* **1987,** *109*, 677–682.

[14] Valeev, E. F.; Coropceanu, V.; da Silva Filho, D. A.; Salman, S.; Brédas, J.-L., Effect of electronic polarization on charge-transport parameters in molecular organic semiconductors. *J. Am. Chem. Soc.* **2006,** *128*, 9882–9886.

[15] Wen, S.-H.; Li, A.; Song, J.; Deng, W.-Q.; Han, K.-L.; Goddard III, W. A., First-principles investigation of anistropic hole mobilities in organic semiconductors. *J. Phys. Chem. B* **2009,**

113, 8813–8819.

[16] Chu, M.; Fan, J.-X.; Yang, S.; Liu, D.; Ng, C. F.; Dong, H.; Ren, A.-M.; Miao, Q., Halogenated Tetraazapentacenes with Electron Mobility as High as 27.8 $cm^2 V^{-1} s^{-1}$ in Solution-Processed n-Channel Organic Thin-Film Transistors. *Adv. Mater.* **2018,** *30*, 1803467.

[17] Gao, M.; Chen, H.; Miao, Q., Synthesis, Structures and Properties of Bis(naphthocyclobuta)pyrenes. *Eur. J. Org. Chem.* **2022,** *2022*, e202101315.
